# Structural basis of lantibiotic recognition by the nisin resistance protein from *Streptococcus agalactiae*

**DOI:** 10.1038/srep18679

**Published:** 2016-01-04

**Authors:** Sakshi Khosa, Benedikt Frieg, Daniel Mulnaes, Diana Kleinschrodt, Astrid Hoeppner, Holger Gohlke, Sander H. J. Smits

**Affiliations:** 1 Institute of Biochemistry, Heinrich Heine University, Universitätsstr. 1, 40225 Düsseldorf, Germany; 2 Institute of Pharmaceutical and Medicinal Chemistry, Heinrich Heine University, Universitätsstr. 1, 40225 Düsseldorf, Germany; 3 Protein Production Facility, Heinrich Heine University, Universitätsstr. 1, 40225 Düsseldorf, Germany; 4 Crystal and X-ray Facility, Heinrich Heine University, Universitätsstr. 1, 40225 Düsseldorf, Germany

## Abstract

Lantibiotics are potent antimicrobial peptides. Nisin is the most prominent member and contains five crucial lanthionine rings. Some clinically relevant bacteria express membrane-associated resistance proteins that proteolytically inactivate nisin. However, substrate recognition and specificity of these proteins is unknown. Here, we report the first three-dimensional structure of a nisin resistance protein from *Streptococcus agalactiae* (*Sa*NSR) at 2.2 Å resolution. It contains an N-terminal helical bundle, and protease cap and core domains. The latter harbors the highly conserved TASSAEM region, which lies in a hydrophobic tunnel formed by all domains. By integrative modeling, mutagenesis studies, and genetic engineering of nisin variants, a model of the *Sa*NSR/nisin complex is generated, revealing that *Sa*NSR recognizes the last C-terminally located lanthionine ring of nisin. This determines the substrate specificity of *Sa*NSR and ensures the exact coordination of the nisin cleavage site at the TASSAEM region.

Antibiotics provide a great advantage in the treatment of infections caused by bacteria such as *Streptococcus pneumoniae* and *Streptococcus agalactiae*. However, due to their widespread use, the number of resistant bacterial strains is increasing^1^, leading to an urgent need for the development of new antibiotics. Several approaches have been taken to identify new antibiotics where naturally occurring compounds are found to be the most promising ones^2^. Here, small antimicrobial peptides such as lantibiotics are excellent candidates because they exhibit high effectivity against various Gram-positive human pathogenic bacteria including *Streptococcus pneumoniae* and several methicillin-resistant *Staphylococcus aureus* (MRSA) strains^3^.

Lantibiotics display antimicrobial activities in the very low nanomolar range^4^,^5^. The anti-infective potency of lantibiotics such as nisin, mutacin, mersacidin and others has been recognized, and several are in the preclinical stages of medical application^6^,^7^. After translation, lantibiotics are modified and contain unusual amino acids such as dehydroalanine (Dha) and dehydrobutyrine (Dhb), which are covalently linked to the side chain of cysteine residues forming the so-called lanthionine rings^8^,^9^. The number as well as the exact location of the lanthionine rings vary within lantibiotics^10^. Lantibiotics have multiple modes of action, of which binding to lipid II, thereby inhibiting cell wall synthesis, and pore formation are the most predominant ones^8^,^11^. Nisin produced by *Lactococcus lactis* (*L. lactis*) is one of more than 50 lantibiotics discovered so far^12^ and is considered to be the role model. Active nisin consists of 34 amino acids and contains five lanthionine-based rings (Supplementary Fig. 1). The first three rings (A-C) are separated from the other two intertwined rings (D-E) by a flexible hinge region. The first two rings are able to bind lipid II^13^; the hinge region and the last two intertwined rings are able to flip into the membrane and create a pore^14^,^15^,^16^.

Due to their multiple modes of action, hardly any resistance against lantibiotics has developed over the past decades. However, some bacterial strains have been reported to be congenitally resistant against nisin^17^ via various mechanisms such as cell wall modifications, biofilm formation or the expression of resistance proteins^18^. For the latter case, a *nsr* gene was identified in the *Streptococcus lactis* subspecies *diacetylactis* (DRC3) that encodes the nisin resistance protein, NSR^17^,^19^. Similar genes were identified in other species^17^,^20^,^21^, including several human pathogenic strains^22^,^23^. NSR is a member of the S41 protease family, specifically the C-terminal processing peptidases (CTPs). NSR from *L. lactis* TS1640 has been shown to degrade nisin by cleaving the peptide bond between MeLan_28_ in ring E and Ser_29_. The resulting nisin_1-28_ fragment displays a significantly lower bactericidal efficacy and reduced affinity towards cellular membranes^24^. Furthermore, the NSR protein from *S. agalactiae* ATCC 13813 induced a 20-fold increased resistance towards nisin when expressed in *L. lactis*^22^.

NSR is localized within an operon comprising five genes, which encode for NSR, a two-component signaling system (NsrRK), and an ABC transporter (NsrFP). When expressed together, these proteins deliver full nisin resistance^22^. Interestingly, similar operon structures were also found to be associated with resistance against other lantibiotics^18^,^23^. These operons resemble (auto)-immunity systems found in lantibiotic producer^10^ strains. Structures of SpaI from *B. subtilis*^25^ conferring resistance against subtilin and MlbQ from the actinomycete *Microbispora* ATCC PTA-5024 conferring resistance against NAI-107^26^ were resolved by NMR. However, no significant sequence identity is found between NSR and SpaI or MlbQ, suggesting a different mechanism for the defense against lantibiotics. Furthermore, most (auto)-immunity proteins do not cleave or manipulate the lantibiotic but rather shield the host’s membrane from being harmed by its lantibiotic^10^,^27^.

The ability of NSR to cleave nisin is impressive because it has been shown for several lantibiotics that they are not easily accessible for protease cleavage^14^. Here, the lanthionine rings are likely causing steric hindrance within the active site of proteases, thereby inhibiting proteolysis. Thus, notwithstanding the recent advances in this field, we still structurally know relatively little about lantibiotic resistance. In particular, the lantibiotic binding site in NSR and the mechanism how substrate specificity is conferred remains elusive. In this study, we report the first structure of a nisin resistance protein, NSR from *S. agalactiae* COH1 (*Sa*NSR). Mutagenesis studies guided by molecular dynamics (MD) simulations reveal that *Sa*NSR recognizes the lanthionine ring closest to the C- terminus of nisin and that this ring binds at one end of the catalytic tunnel, thereby determining the substrate specificity and ensuring the exact coordination of the nisin cleavage site at the catalytic site region.

## Results

### Crystal structure of *Sa*NSR

Nisin has been shown to be quite resistant against proteolytic digestion in general^14^, supposedly due to the presence of lanthionine rings. Therefore, it is intriguing to understand the proteolytic resistance mechanism mediated by NSRs. To obtain a molecular view on this mechanism, we solved the structure of *Sa*NSR by X-ray crystallography.

Through sequence analyses, it was predicted that the first 30 amino acids encode for a transmembrane helix^28^. We deleted this N-terminal transmembrane helix and included a His_8_-tag for purification purposes, resulting in soluble expression of *Sa*NSR. After over-expression, two-step purification yielded 5 mg of pure *Sa*NSR protein per liter of cell culture (Supplementary Fig. 2a). *Sa*NSR is a monomer in solution as determined by multiple angle light scattering (MALS) (Supplementary Fig. 2b). *Sa*NSR protein was crystallized and cubic crystals were obtained that diffracted up to 2.2 Å resolution^29^. We solved the structure by Single Anomalous Dispersion (SAD) phasing, using crystals of selenomethionine-substituted protein (data and refinement statistics are shown in Table 1).

The asymmetric unit (Supplementary Fig. 2c) contained four copies of *Sa*NSR that were virtually identical (root mean square deviation (RMSD) between the monomers = 0.15–0.5 Å over 300 amino acids). Therefore, the overall structure is described only for monomer A. The entire sequence of *Sa*NSR could be fitted into the electron density, with the exception of the N-terminal His_8_-tag that was disordered. The R_work_ and R_free_ values after refinement were 0.19 and 0.24, respectively.

**Figure 1:**
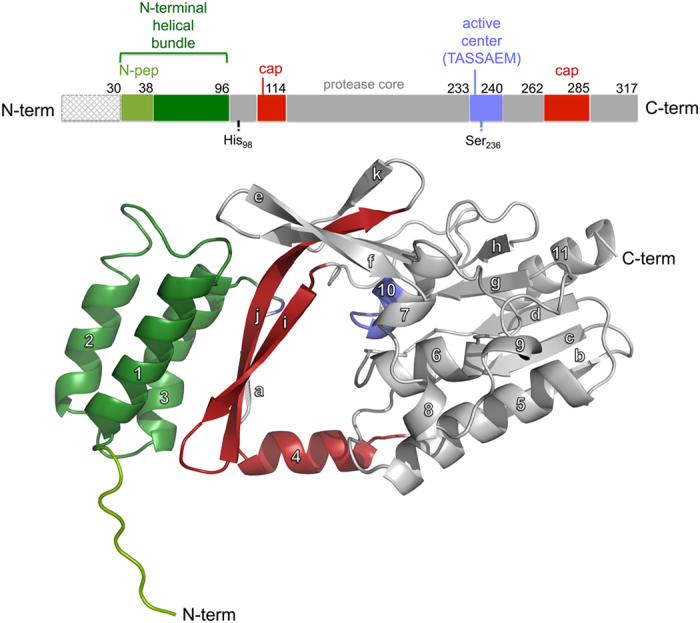
Structural architecture of the *Sa*NSR monomer. (**a**) Schematic illustration of the domain organization of *Sa*NSR indicating the domain borders and catalytically important residues (His_98_ and Ser_236_). (**b**) The overall structure of a *Sa*NSR monomer in a cartoon representation. The N-terminal helical bundle is depicted in green where the light green region represents the N-pep. The protease cap and core domains are highlighted in red and grey, respectively. The catalytically important residues and the highly conserved “TASSAEM” region are depicted in blue.

**Figure 2:**
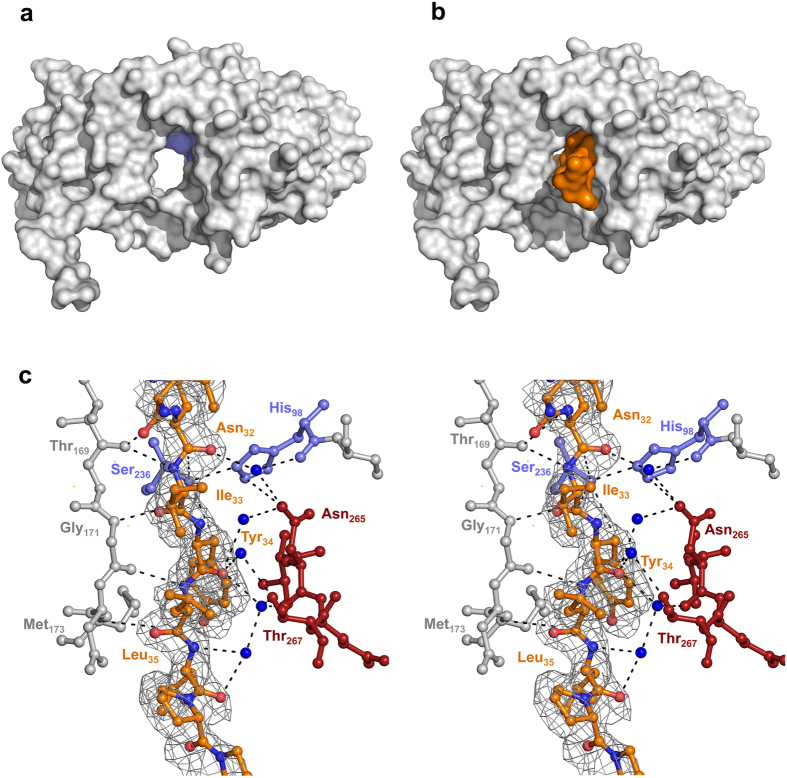
Surface representation of *Sa*NSR. (**a**) The surface representation of *Sa*NSR in white, highlighting the tunnel localized in between the protease cap and the core domain. The TASSAEM motif is colored in blue. (**b**) Surface representation of *Sa*NSR with bound N-pep (colored in orange). (**c**) Stereo view on the active site architecture of *Sa*NSR highlighting the N-pep that is bound within the tunnel as ball and stick representation. The corresponding 2F_o_F_c_ omit electron density map is calculated at 2.2 Å and contoured at 1.0 σ. The water mediated interactions of N-pep (colored in orange) with residues of the protease cap (depicted in red) and the direct interactions with the residues of the protease core (grey color) are shown.

A *Sa*NSR monomer (Fig. 1) consists of eleven helices (α_1_-α_11_) and eleven β-strands (β_a_-β_k_), which form three domains: an N-terminal helical bundle and two protease subdomains. Altogether, these domains form a hydrophobic tunnel of ~10 Å width (Fig. 1b), which could very well harbor the nisin molecule. The N-terminal helical bundle (Fig. 1b, represented in green) comprises 65 amino acid residues (Lys_31_-Gly_96_), which form helices α_1_-α_3_. This domain ends in a triple glycine motif (_94_GGG_96_) before entering the protease cap domain (Fig. 1b, represented in red). The protease cap domain consists of helix α_4_ and a β-hairpin structure formed by strands β_i-j_. The protease cap forms a lid-like structure above the tunnel. The third domain is the so-called protease core domain (Fig. 1b, represented in grey), which adopts a ‘protease fold’ domain as observed in other S41 peptidases^30^,^31^,^32^. The protease core domain is formed by six strands β_b_-β_g_ and five helices α_5_-α_9_. It contains the highly conserved TASSAEM region that harbors the previously identified catalytically active serine at position 236^22^ (Fig. 1, represented in blue; Supplementary Fig. 3). The TASSAEM region lies in the tunnel between the two protease subdomains (Fig. 2a).

### N-terminal helical bundle

A comparison of the N-terminal helical bundle with all available entries in the Protein Data Bank was performed using the Dali server^33^. The Dali server identifies similarities in 3D structures irrespective of sequence similarities. A structurally similar helical bundle has been identified in the human Factor H (Z-score of 5.2), which is responsible for tight binding of the pneumococcal protein virulence factor CbpA (choline-binding protein A)^34^. Furthermore, a similar helical bundle is present at the C-terminus of the 70 kDa human heat shock protein (HSP70) (Z-score of 5.0). This region is responsible for causing a structural switch during HSP70 allosteric activation, which is important for maintaining a proper conformation of the protein for binding to the J-domain and ATPase activity purposes^35^. Finally, a dynamic helical region is present at the N-termini of staphylococcal complement inhibitors (SCINs) (Z-score of 4.7), which is responsible for binding to the substrate C3b and is also necessary for the formation of higher order complexes of C3b, which blocks phagocytosis^36^. While these findings suggest that a certain degree of mobility of the found helical bundles is required for function, in three replicates of molecular dynamics (MD) simulations of a monomer of *Sa*NSR (termed NSR_Apo_; see online methods for details), each of 500 ns length, the N-terminal helical bundle is rather immobile with respect to the protease core and cap domain (backbone root mean square fluctuations (RMSF) <2.5 Å; Supplementary Fig. 4a).

### N-pep bound to *Sa*NSR

In the crystal structure, the hydrophobic tunnel is filled with the N-terminal residues _31_KNIYLLPP_38_ of a neighboring *Sa*NSR molecule (termed N-pep; Fig. 2b; shown in light green in Fig. 1). N-pep is predominantly bound to *Sa*NSR via direct backbone interactions to amino acids _167_NNTGGN_172_ of β-strand β_d_, which is part of the protease core domain and is structurally located on the opposite site of the TASSAEM sequence motif (Fig. 2c). In addition, N-pep is stabilized via water-mediated hydrogen bonds between backbone atoms of Asn_32_, Tyr_34_, Leu_35_ and residues Asn_265_ and Thr_267_ of the protease cap domain (Fig. 2c). The presence of N-pep within the tunnel is clearly an induced artifact of the crystallization procedure, since in the full-length *Sa*NSR protein, another 30 amino acids are attached at the N-terminus of the N-pep sequence that form a transmembrane helix. Yet, during MD simulations of 500 ns length of a *Sa*NSR monomer complexed with N-pep (termed NSR_Tail_; see online methods for details), N-pep remains stably bound within the hydrophobic tunnel (mean backbone RMSD < 1.6 Å; Supplementary Fig. 4b). The predominance of backbone interactions of N-pep with the protease core and cap domains could explain why N-pep binds into the putative binding region of nisin despite its sequence being very dissimilar to the one of nisin.

### Substructures of nisin determining its molecular recognition

**Figure 3:**
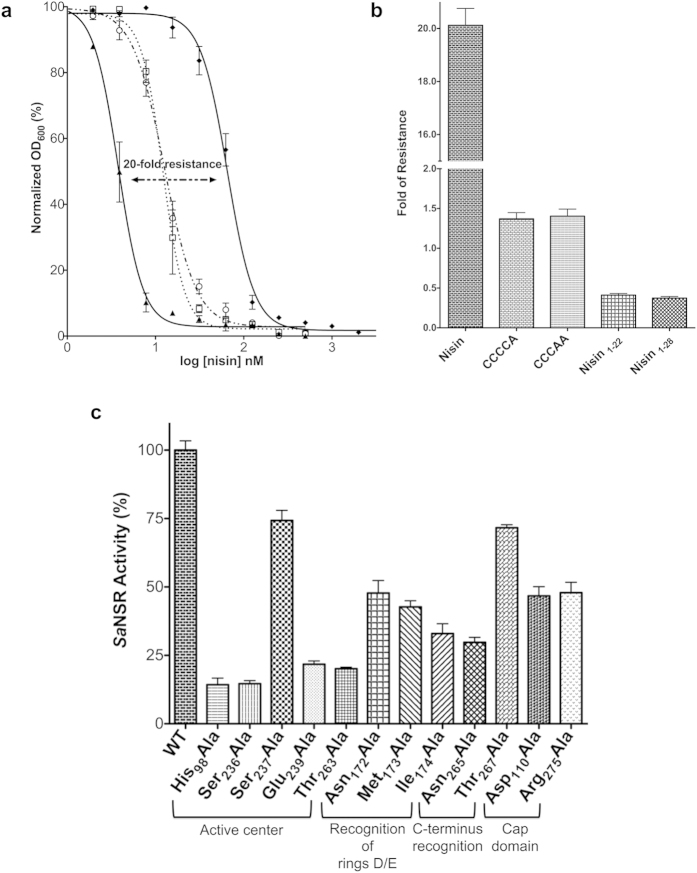
Influence of wild type *Sa*NSR and its mutations against nisin and its variants. (**a**) Growth inhibition experiment of *Sa*NSR with nisin. The activity of *Sa*NSR is determined using the *L. lactis* NZ9000 strain, where the plasmid encoding the *Sa*NSR wildtype and the mutations were transformed, and the IC_50_ against nisin was determined. As a control, the empty vector was transformed and used in the IC_50_ study (termed NZ9000Erm). Black lines represent the NZ9000Erm (filled **Δ**) and NZ9000-*Sa*NSR (♦) strains, respectively. The black dotted lines represent the NZ9000-*Sa*NSR-His_98_Ala (☐) and NZ9000-*Sa*NSR-Ser_236_Ala (Ο) strains. The data were fitted and evaluated as described in ref. 51. The difference in the growth exhibited by the strains was used to calculate the percentage of activity. Each experiment was performed at least in triplicates. (**b**) Graphical representation of the fold of resistance exhibited by *Sa*NSR with nisin and different nisin variants (CCCCA, CCCAA, nisin_1-22_ and nisin_1-28_). The NZ9000Erm and NZ9000*Sa*NSR strains were used to determine the activity of all the nisin variants. The error bars indicate the standard error of at least three independent experiments. (**c**) The activity of *Sa*NSR and its mutations is determined using the *L. lactis* NZ9000 strain. A normalization of the IC_50_ values were done by setting the values exhibited by the empty vector (NZ9000Erm) and NZ9000*Sa*NSR to 0% and 100%, respectively. The error bars indicate the standard error of at least three independent experiments.

To investigate the substrate specificity of *Sa*NSR and determine substructures of nisin important for its recognition by the protein, we used different nisin variants. In order to test the influence of rings D and E located next to the cleavage site of nisin, we genetically replaced the last or the last two cysteine(s) in nisin by alanine, resulting in the expression of active nisin containing only rings A-D (termed CCCCA) or A-C (termed CCCAA), respectively^27^. Similarly, we removed the last six amino acids of nisin (termed nisin_1-28_), resulting in the product of the proteolysis reaction mediated by *Sa*NSR. Furthermore, a truncated variant (nisin_1-22_) was expressed, which contained the rings A-C but lacked the rest of the C-terminus of nisin^27^. Since all variants show a different activity against the nisin sensitive *L. lactis* NZ9000Erm strain (Supplementary Table 1a), we analyzed them with respect to the fold of resistance mediated by the expression of the *Sa*NSR protein in the NZ9000*Sa*NSR strain. From current and previous work, it is known that *Sa*NSR confers a 20-fold increased resistance against wildtype nisin (Fig. 3a)^22^.

For CCCCA as well as CCCAA, the resistance mediated by *Sa*NSR decreased to roughly 1.4–1.7 fold when comparing the IC_50_ values of the different strains (Fig. 3b; Supplementary Table 1a). For the truncated variants, nisin_1-22_ and nisin_1-28_, no resistance was observed anymore as the IC_50_ values dropped to the levels observed for the NZ9000Erm strain. Thus, the lanthionine ring E is clearly important for the recognition by *Sa*NSR.

### Structural model of nisin binding to *Sa*NSR

Despite intensive trials, we were not successful in obtaining a crystal structure of a *Sa*NSR/nisin complex. Thus, we resorted to generating a structural model by integrative modeling and validating it by mutagenesis studies. Initially, we structurally aligned the backbone of residues 31–36 of nisin to the backbone of N-pep such that the nisin cleavage site between ring E and Ser_29_ was oriented towards the catalytically active Ser_236_ in *Sa*NSR. Rings D and E were then manually placed in three orientations at the tunnel entrances such that they showed good complementarity with the *Sa*NSR surface. This resulted in three structural models of *Sa*NSR/nisin complexes, two (termed NSR_Nisin,1_, NSR_Nisin,2_) where rings D and E are located close to Asn_172_, Met_173_, and Ile_174_, and one (termed NSR_Nisin,3_) where nisin is oriented oppositely with respect to the tunnel axis such that Tyr_261_ stacks onto rings D and E (Supplementary Fig. 5a). The three models were subjected to MD simulations^37^ of 500 ns length, with three replicate simulations each.

**Figure 4:**
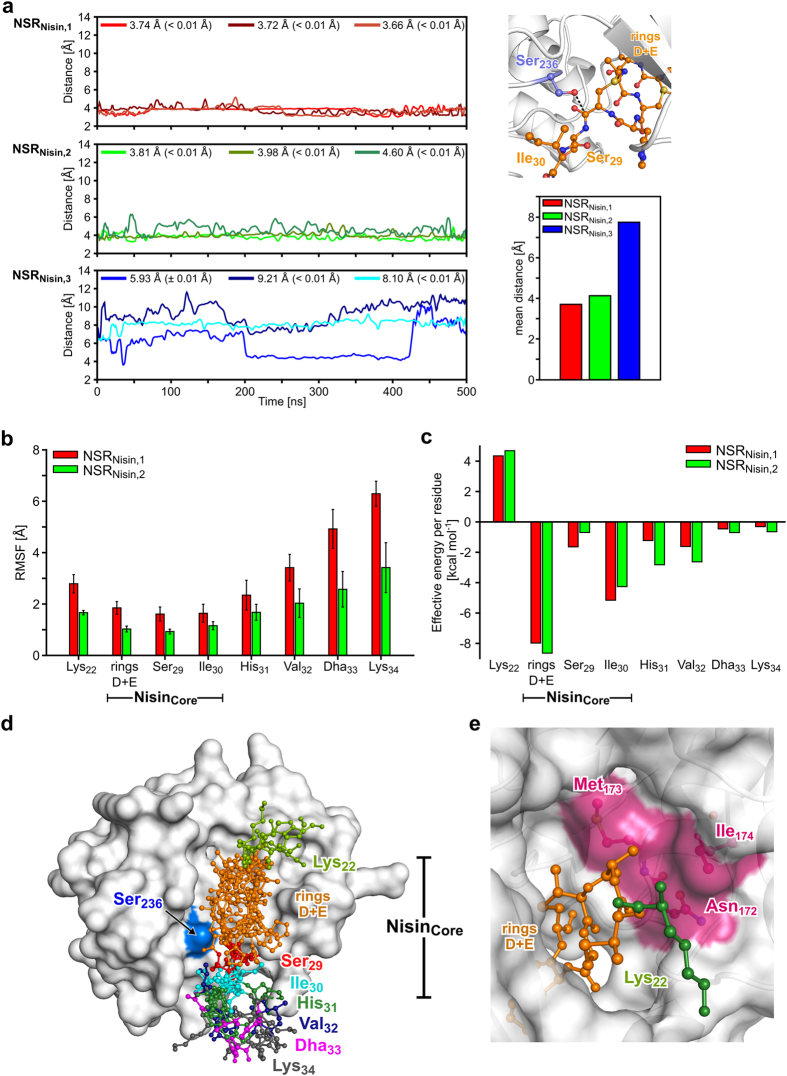
Structural and energetic analysis of MD simulations of *Sa*NSR/nisin model complexes. (**a**) Distance between the side chain oxygen of Ser_236_ and the carbonyl carbon of ring E at the nisin cleavage site (black dotted line in the upper right panel) in NSR_Nisin, {1, 2, 3}_ during 500 ns of MD simulations; lines were smoothed by cubic splines. Mean values and standard error of the mean (SEM; in parentheses) are shown in the legend. The mean distance over all three MD simulations is shown in the lower right panel (SEM < 0.1 Å and not shown). (**b**) Mean backbone RMSF (SEM indicated as error bars) for NSR_Nisin, {1, 2}_ models over three trajectories each of 500 ns length. Rings D and E, Ser_29_, and Ile_30_ compose the Nisin_Core_. (**c**) Mean effective binding energy per residue for NSR_Nisin,{1, 2}_ models. Error bars indicate SEM over three trajectories. (**d**) Superimposition of six close-to-average structures (based on the backbone RMSD) of nisin (ball-and-stick models each colored differently), extracted from three independent MD simulations each of NSR_Nisin,1_ and NSR_Nisin,2_, within the tunnel of *Sa*NSR (white surface representation). Ser_236_ of the catalytic dyad is colored in blue. For clarity, the N-terminal helical bundle and part of the cap region of *Sa*NSR have been omitted. (**e**) Representative nisin model (orange and green ball-and-stick model) within the tunnel of *Sa*NSR (white cartoon representation with transparent surface). Residues Asn_172_, Met_173_, and Ile_174_ that bind to rings D and E are colored in magenta.

The average distances between the side chain oxygen of Ser_236_, previously identified as the catalytically active serine^22^, and the carbonyl carbon of Ser_29_ at the nisin cleavage site^24^ are 3.71 Å, 4.13 Å, and 7.74 Å for NSR_Nisin,1_, NSR_Nisin,2_ and NSR_Nisin,3_, respectively (Fig. 4a). This strongly indicates that a nucleophilic attack of the side chain of Ser_236_ at the nisin cleavage site as a first step in the catalytic mechanism^24^ is possible for the first two models but not for the third, suggesting that NSR_Nisin,1_ and NSR_Nisin,2_ represent the most likely orientation of nisin within the *Sa*NSR tunnel. Thus, we focused further analyses on the NSR_Nisin,1_ and NSR_Nisin,2_ models.

Visual inspection of the MD trajectories and computations of the backbone RMSF identified residues Lys_22_, His_31_, Val_32_, Dha_33_, and Lys_34_ of nisin as highly mobile (RMSF values up to 6.39 Å ± 0.49 Å) (Fig. 4b). In contrast, the core region (Nisin_Core_) composed of the rings D and E, and residues Ser_29_ and Ile_30_ revealed RMSF values <1.85 Å ± 0.24 Å (Fig. 4b) suggesting a tightly bound Nisin_Core_ region. This was corroborated by a per-residue decomposition of effective binding energies computed by the MM-PBSA approach^38^. Here, rings D and E (treated as one residue in the energy decomposition) and Ile_30_ are identified as essential for nisin binding (residue-wise effective binding energies in the range from −4.26 kcal mol^−1^ to -8.63 kcal mol^−1^) (Fig. 4c). In contrast, for Ser_29_, a smaller contribution to the effective binding energy of -1.64 kcal mol^−1^ (−0.70 kcal mol^−1^) for *Sa*NSR_Nisin,1_ (*Sa*NSR_Nisin,2_) was found (Fig. 4c). Overall, this suggests that the rings D and E as well as Ile_30_ form a binding motive, that way ensuring that also Ser_29_ at the nisin cleavage site is correctly positioned within the catalytic site.

In Figure 4d, a representative set of six nisin structures within the *Sa*NSR tunnel is shown. For this, the structure with the smallest backbone RMSD to the average structure was extracted from each of the NSR_Nisin,1_ and NSR_Nisin,2_ MD trajectories. The set shows that the location and orientation of rings D and E, Ser_29_, and Ile_30_ agree well in all cases, with RMSD values with respect to the average structure for the Nisin_Core_ ranging from 0.80 Å to 2.27 Å (Supplementary Fig. 5b). Thus, both NSR_Nisin,1_ and NSR_Nisin,2_ models were considered equivalent and used to identify residues in *Sa*NSR important for catalysis and nisin binding for mutagenesis studies. The remaining residues of nisin show large structural deviations, in agreement with the above analyses (Supplementary Fig. 5b, Fig. 4b,c).

### The TASSAEM region and His_98_ form the active site

The NSR superfamily contains a highly conserved sequence motif “TASSAEM” (Supplementary Fig. 3) located at the rear end of the protease core domain. Within this TASSAEM region, Ser_236_ has been previously identified as the catalytically active serine^22^. This serine is in close proximity to the strictly conserved His_98_ residue, which is localized at the end of the N-terminal helical bundle directly next to the _94_GGG_96_ motif (Fig. 1) and is in hydrogen bonds distance with the side chain of Ser_236_. In the NSR_Nisin,1_ and NSR_Nisin,2_ MD simulations, hydrogen bonds were found in up to ~23% of all conformations (Supplementary Fig. 6a), which indicates that both residues likely interact also in the nisin-bound state. Based on the interactions of Ser_236_ and His_98_ and the absence of any other lysine or aspartate residue localized nearby, we presume that *Sa*NSR acts via a catalytic dyad mechanism as observed for some other serine proteases^39^,^40^. The NZ9000*Sa*NSR-Ser_236_Ala strain displayed a low background activity as observed by an IC_50_ value of 12.6 ± 0.7 nM (Supplementary Table 1b), which relates to a *Sa*NSR residual activity of 14% (Fig. 3c). The His_98_Ala mutation displayed a similar IC_50_ value of 12.3 ± 1.5 nM and a residual activity of 14% (Fig. 3c, Supplementary Table 1a). The residual activity displayed by both variants is likely due to the binding of nisin to that particular *Sa*NSR variant such that a higher concentration of nisin is required to kill the corresponding nisin sensitive NZ9000Erm *L. lactis* strain.

Within the TASSAEM sequence, a second serine residue, Ser_237_, is present. In the NSR_Nisin,1_ and NSR_Nisin,2_ MD simulations, the mean distance between the side chain oxygen and the carbonyl carbon of ring E is <5.7 Å (Supplementary Fig. 6b). However, the distance to the *δ*-nitrogen of His_98_ is >9 Å (Supplementary Fig. 6c), and no hydrogen bonds were detected between both residues, making a proton shift between Ser_237_ and His_98_ unlikely. Instead, we observed hydrogen bond formation between the side chain of Ser_237_ and the backbone of Gly_171_ of the protease core in at least 46% of the conformations that may be relevant for nisin recognition (see section “Residues involved in nisin recognition and *Sa*NSR specificity”) but not for catalytic activity. Thus, Ser_237_ is not expected to be involved in the catalytic mechanism. In accordance, a Ser_237_Ala mutation does not have a pronounced effect on the activity of *Sa*NSR (residual activity 74%; see Fig. 3c, Supplementary Table 1b).

The next residue in the TASSAEM motif is Glu_239_, which is pointing away from the active site. In the crystal structure, the Glu_239_ side chain interacts with backbone atoms of Gly_260_ and Tyr_261_ via hydrogen bonds, and during the NSR_Nisin,1_ and NSR_Nisin,2_ MD simulations this interaction is present in at least 82% of all conformations (Supplementary Fig. 6a). Additionally, we found hydrogen bond interactions between Glu_239_ and Ser_236_ in at least 25% of the cases (Supplementary Fig. 6a). These interactions are likely important for the correct positioning of the TASSAEM region. This is in line with the drastically lowered activity of the Glu_239_Ala mutant (IC_50_ value of 17.1 ± 0.7 nM; residual activity of 22%; Fig. 3c, Supplementary Table 1b). Furthermore, we found stabilizing hydrogen bonds between Thr_263_ and His_98_ in up to ~28%, and between Asn_265_ and His_98_ in up to ~20% of all cases (Supplementary Fig. 6a). These interactions likely ensure a correct orientation of His_98_ as the mutations Thr_263_Ala and Asn_265_Ala decreased the residual activities of *Sa*NSR to 20% and 30%, respectively, (Fig. 3c) with associated IC_50_ values of 16.0 ± 0.3 and 22.1 ± 1.1 nM (Supplementary Table 1b). Taken together, the TASSAEM sequence is crucial for the activity of *Sa*NSR and contains the catalytically active serine as well as a glutamic acid residue, which is likely responsible for a correct positioning of the TASSAEM helix.

### Residues involved in nisin recognition and *Sa*NSR specificity

Next, we investigated nisin recognition by *Sa*NSR. Residue-wise effective binding energies were computed for both the NSR_Nisin,1_ and NSR_Nisin,2_ MD trajectories to identify *Sa*NSR residues likely to be important for nisin binding (Supplementary Fig. 7a). Considering energies < −0.8 kcal mol^−1^ resulted in seven candidates (Leu_102_, Leu_137_, Asn_172_, Met_173_, Ile_174_, Glu_266_, Ala_277_). Our model (Fig. 4d) suggests that the hydrophobic residues Leu_102_, Leu_137_, Met_173_, Ile_174_, Ala_277_ and the polar/charged ones Asn_172_ and Glu_266_ bind to rings D and E in nisin. Asn_172_, Met_173_, and Ile_174_ form a pocket that harbors rings D and E in our model (Fig. 4e). The Asn_172_Ala mutant displayed an activity of 47% (IC_50_ value of 33.5 ± 2.9 nM) (Fig. 3c, Supplementary Table 1b). Furthermore, when mutating the strictly conserved Met_173_ residue (Supplementary Fig. 3), a reduced activity of 42% compared to the wild type value was observed (IC_50_ value of 30.3 ± 1.4 nM). Additionally, the Ile_174_Ala mutant exhibited an activity of 33% (IC_50_ value of 24.1 ± 2.2 nM) (Fig. 3c, Supplementary Table 1b).

Moreover, we found hydrogen bonds between backbone atoms of Thr_169_ and Gly_171_ with the Nisin_Core_ residues (Supplementary Fig. 7b). These interactions are reminiscent to those found for N-pep (Fig. 2c) and likely ensure a proper placement of the Nisin_Core_ within the binding site. Additional stabilizing hydrogen bonds were observed between Asn_168_ and Gly_170_ (Supplementary Fig. 7b), which could contribute to nisin binding indirectly. A similar indirect effect was found for Glu_266_, for which we observed salt-bridge formation with Arg_54_ from the N-terminal helical bundle (Supplementary Fig. 7c; mean distance <3.4 Å). We also found water-mediated hydrogen bonds between backbone atoms of rings D and E, and Asn_265_ and Thr_267_, respectively (Supplementary Fig. 7d), again mimicking what was observed for the bound N-pep (Fig. 2c). Accordingly, the mutations Asn_265_Ala (see above) and Thr_267_Ala decreased the residual activity of *Sa*NSR to 30% and 71%, respectively, (Fig. 3c) with associated IC_50_ values of 22.1 ± 1.1 and 48.5 ± 0.6 nM (Supplementary Table 1b).

### Role of the protease cap domain in *Sa*NSR

Other S41 peptidases also contain a protease cap domain comprising a helix and a β-hairpin structure, where the helix appears to open and close depending on the presence of the peptide substrate: once a peptide is bound, the cap closes and seals the active site. As such, the protease CtpB from *Bacillus subtilis* has been crystallized in an open and closed state with the helix of the protease cap moving by 10–15 Å towards the active site once the peptide was bound^30^. In *Sa*NSR, helix α_4_ (_103_SKETVRRDTLDS_114_) was identified as the protease cap helix, localized directly after the N-terminal helical bundle. Out of all residues of this helix, only the side chain of Asp_110_ is intruding into the tunnel, which neither forms an interaction to N-pep in the crystal structure nor in the NSR_Tail_ MD simulations. This suggests that the protease cap is not adopting a fully closed state, rather an intermediate state. MD simulations show a salt-bridge formation between Asp_110_ and Arg_275_ of the protease cap domain for both NSR_Nisin1,2_ models (Supplementary Fig. 8a). In those cases where the salt-bridge formation is weak (mean distance is > 10 Å), a loss of the secondary structures of helix α_4_ is observed (Supplementary Fig. 8b). The Asp_110_Ala mutant of *Sa*NSR is still active although with a lower IC_50_ value of 32.8 ± 2.1 nM (residual activity of 46%; Fig. 3c, Supplementary Table 1b). The Arg_275_Ala mutant revealed an identical IC_50_ value of 33.6 ± 2.3 nM (residual activity of 48%). Taken together, this suggests that a proper secondary structure of helix α_4_ is required for *Sa*NSR function, and that Asp_110_ contributes to the stability of the secondary structure.

## Discussion

**Figure 5:**
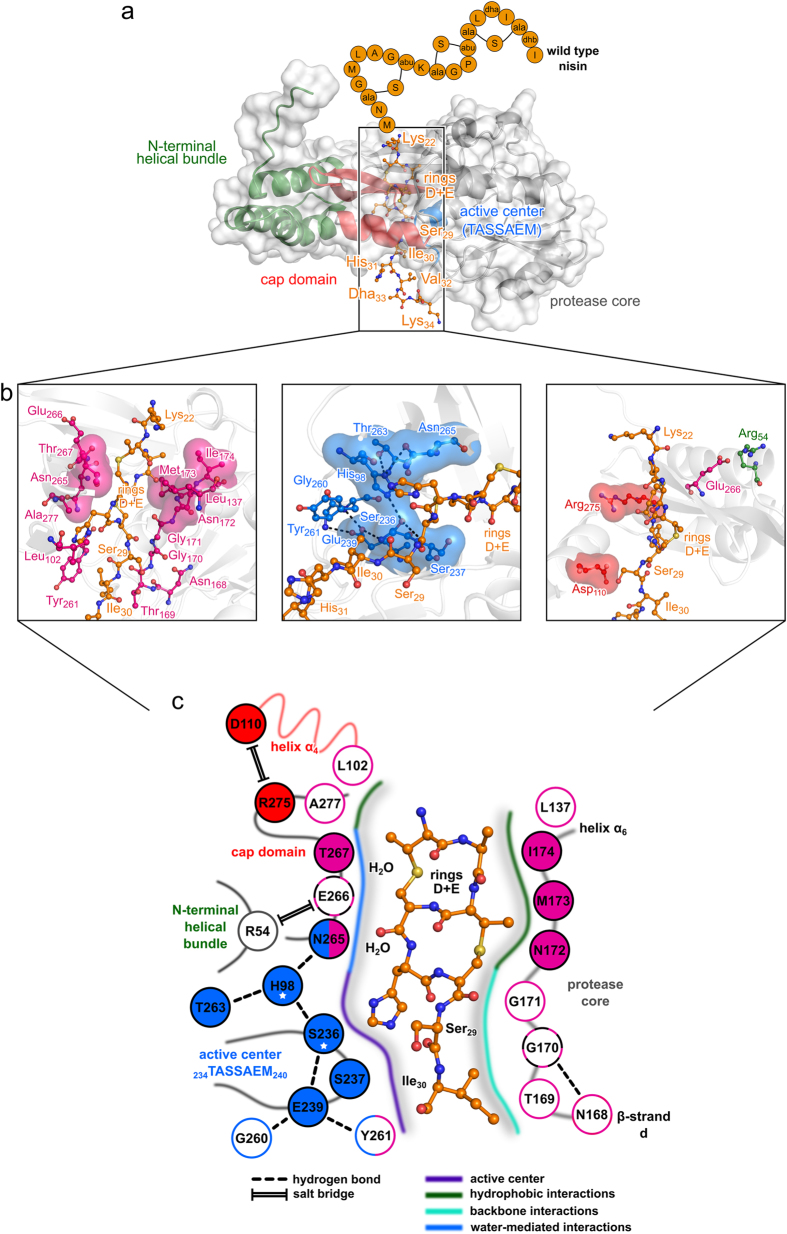
Nisin/*Sa*NSR binding model. (**a**) Representative structure of nisin (residues 22-34; extracted from the NSR_Nisin,1_ model) bound to the crystal structure of *Sa*NSR (cartoon representation with transparent surface; each domain is colored differently). Orange spheres with one-letter/three letter amino acid code indicate nisin residues 1–21 not considered for modeling studies (abbreviations: abu = aminobutyric acid; dha = dehydroalanine; dhb = dehydrobutyrine; ala-S-X = lanthionine derivatives). (**b**) Close up view of nisin binding to *Sa*NSR residues important for nisin recognition (left), residues with catalytic function (middle), and residues with a regulatory function (right). Amino acids of interest are depicted as ball-and-stick model; residues for which experimental data is reported in this study are, additionally, shown in transparent surface representation. (**c**) Schematic representation of the Nisin_core_ bound to *Sa*NSR residues (residue numbers according to the crystal structure described here). Residues that compose the catalytic site are colored in blue, residues that contribute to nisin binding in magenta, residues that have an indirect effect on binding in black-magenta, and residues with a supposedly regulatory function in *Sa*NSR in red. For residues with colored background, *Sa*NSR activity information for alanine mutants is available (see Fig. 3c). Residues marked with a star form the catalytic dyad. In panels a, b, and c, the nisin structure is depicted as orange ball-and-stick model.

The present study reveals that the lanthionine ring E of nisin determines substrate specificity of the nisin resistance protein (NSR) and contributes to the coordination of the nisin cleavage site at the catalytic center. These results are based on the first structure of a nisin resistance protein from *S. agalactiae* COH1 (*Sa*NSR) at 2.2 Å resolution and subsequent integrative modeling and mutagenesis studies. The *Sa*NSR structure consists of an N-terminal helical bundle, a protease cap domain, and a protease core domain (Fig. 1). The core domain harbors the highly conserved TASSAEM motif, which contains the catalytically important Ser_236_ residue, in a hydrophobic tunnel formed by all three domains. In this tunnel, an N-terminal peptide from another *Sa*NSR protomer (N-pep) in the asymmetric unit is bound predominantly by direct and water-mediated backbone hydrogen bonds (Fig. 2). A very similar binding pattern is found for the C-terminal lanthionine rings D and E, and residues Ser_29_, and Ile_30_ of nisin in our model of the *Sa*NSR/nisin complex (Fig. 5a,b; Supplementary Fig. 7d). According to this model, lanthionine ring E binds at one end of the hydrophobic tunnel (Fig. 5a,b) and ensures the exact coordination of the nisin cleavage site at the highly conserved TASSAEM region (Fig. 5a,b).

In contrast to some other C-terminal processing proteases^30^,^32^, the active center of *Sa*NSR consists of a catalytic dyad formed by residues Ser_236_^22^, which is part of the TASSAEM motif, and His_98_ as determined by mutational analysis and also described for some other proteases^41^ (Fig. 5a–c). Mutational analysis and geometric parameters in the crystal structure and during MD simulations exclude that the neighboring Ser_237_ participates in the catalytic step. Residues Glu_239_, Gly_260_, Tyr_261_ and Thr_263_, form hydrogen bonds with either Ser_236_ or His_98_ during all-atom MD simulations of the *Sa*NSR/nisin complexes (Supplementary Fig. 6a, Figure 5a–c) and, thus, likely stabilize the catalytic residues, as also indicated by alanine mutations of these residues that lead to a decrease in *Sa*NSR activity (Fig. 3c).

Since all our efforts to obtain crystals of *Sa*NSR with bound nisin were unsuccessful, we generated a model (Fig. 5a–c) of the *Sa*NSR/nisin complex by integrative modeling and subsequent site-directed mutagenesis studies and activity measurements for validation. The modeling step was guided by exploiting the knowledge on the location of N-pep in the *Sa*NSR crystal structure as well as on the substructures of nisin determining its molecular recognition. As to the latter, we focused on the C-terminus of nisin (nisin_22-34_) where NSR from *L. lactis* TS1640 has been shown to cleave^24^. As a result, nisin variants in which the bulky lanthionine rings D and E, or only E, were replaced by a linear sequence (CCCCA, CCCAA) showed a large drop in the fold of resistance comparable to those exhibited when the last 12 or 6 residues of nisin (nisin_1-22_, nisin_1-28_) were missing (Fig. 3b). These results demonstrated that ring E is essential for nisin recognition by *Sa*NSR.

Initial models of *Sa*NSR/nisin complexes were generated in which the linear, C-terminal sequence (sequence Lys_22_ - Lys_34_) were placed at the location of the backbone trace of N-pep and where rings D and E showed a good complementarity with the *Sa*NSR surface at the tunnel entrance. We considered that no *a priori* knowledge on the direction of nisin with respect to the tunnel axis was available by generating models with both possible directions. By subsequent all-atom MD simulations, we could exclude one of the possibilities (NSR_Nisin,3_) as in this case the distance between Ser_236_ and the nisin cleavage site was too large as to allow for a nucleophilic attack of the serine side chain (Fig. 4a). In contrast, for the other direction (NSR_Nisin,1,_ NSR_Nisin,2_), such an attack is very likely according to distances that are only slightly larger than the sum of van der Waals radii of oxygen and carbon. This model of a *Sa*NSR/nisin complex is further supported by rather immobile residues of the core region of nisin (rings D and E, Ser_29_ and Ile_30_), which is considered to facilitate a nucleophilic attack, in contrast to the more mobile C-terminal residues 31-34 (Fig. 4b), and by a residue-wise decomposition of the effective binding energy, which identified rings D and E as well as Ile_30_ as major contributors to the binding affinity (Fig. 4c).

The model (Fig. 5a–c) reveals that *Sa*NSR binding to rings D and E of nisin is dominated by hydrophobic interactions (Fig. 5b,c). Within the protease core Asn_172_, Met_173_, and Ile_174_ form a pocket that harbors both rings D and E (Figs 4e,5b). In agreement with this model, mutation of these residues reduces the activity of *Sa*NSR. Furthermore, water-mediated hydrogen bonds between backbone atoms of rings D and E and side chains of Asn_265_ and Thr_267_, respectively, were identified, mimicking interactions with N-pep. Asn_265_Ala and Thr_267_Ala mutations decreased the residual activity of *Sa*NSR (Fig. 3c). Finally, along the tunnel, hydrogen bonds between backbone atoms of Thr_169_ and Gly_171_ of *Sa*NSR with Ser_29_ and Ile_30_ of nisin were found (Fig. 5b,c; Supplementary Fig. 7b), which likely contribute towards the correct orientation of the nisin cleavage site at the catalytic center and are again reminiscent of interactions observed for N-pep in the crystal structure.

N-pep and the C-terminus of nisin are not similar on the amino acid level. Together with the above findings of similar interactions along the tunnel between backbone atoms of *Sa*NSR and the two peptides, respectively, this suggests that the tunnel’s role in peptide binding is not to confer substrate specificity but rather to “rope in” the peptide while establishing these interactions. In the case of nisin, this “roping in” is stopped when the lanthionine ring E starts interacting with *Sa*NSR, thereby acting as a plug on the tunnel (Fig. 5c). These interactions are highly relevant for the molecular recognition of nisin and the substrate specificity of *Sa*NSR, as shown by a decrease in the fold of resistance for the nisin variants CCCCA and CCCAA (Fig. 3b) and a decrease in the activity of *Sa*NSR mutants Asn_172_Ala, Met_173_Ala, and Ile_174_Ala (Fig. 3c). In addition, rings D and E are highly likely relevant for a proper placement of the nisin cleavage site with respect to the catalytic Ser_236_, as only with nisin a distance to this residue compatible with a nucleophilic attack and, simultaneously, hydrogen bonds with His_98_ are found in the MD simulations. In contrast, during MD simulations of NSR_Tail_, no hydrogen bond formation between Ser_236_ and His_98_ was detected. This may explain why N-pep binds to *Sa*NSR but is not cleaved.

Previously, an “inhibiting role” of lanthionine rings has been recognized in that they protect lantibiotics from degradation by standard proteases^42^, likely because of their bulky 3D structure which prevents a proper placement in the substrate binding regions of proteases evolved to cleave linear peptides. In turn, the findings in this study for the first time reveal a significant “fostering role” of the lanthionine rings D and E in nisin for the highly specific cleavage of this lantibiotic by *Sa*NSR. These findings and our structural model of the *Sa*NSR/nisin complex open up a new avenue in the understanding of lantibiotic resistance by human pathogens. They may also facilitate the development of therapeutics to overcome nisin resistance.

## Methods

### Cloning, expression and purification of *Sa*NSR

The *nsr* gene from *Streptococcus agalactiae* COH1 was cloned into pET28b and purified as previously described. For details see Supplementary Information.

### Multiple angle light scattering

For HPLC-MALS analysis, a Bio SEC-5 HPLC column (Agilent Technologies Deutschland GmbH, Böblingen, Germany) with a pore size of 300 Å was equilibrated with 25 mM MES pH 6.0, 150 mM NaCl for HPLC using a system from Agilent Technologies connected to a triple-angle light-scattering detector (miniDAWN TREOS, Wyatt Technology Europe GmbH, Dernbach, Germany) followed by a differential refractive index detector (OPTILab T-rEX, Wyatt Technology). Typically, 100 μl of purified *Sa*NSR (2.0 mg/ml) was loaded onto the Bio SEC-5 HPLC column, and the obtained data were analyzed with the ASTRA software package (Wyatt Technology).

### Crystallization, data collection and structure determination of *Sa*NSR

Crystals were obtained and optimized as described in the Supplementary Information. X-ray diffraction data were collected at the ID23eh2 or ID29 beamlines of the European Synchrotron Radiation Facility (ESRF), Grenoble. All the data sets were processed and scaled using XDS and XSCALE software package^43^. Data sets from native crystals were collected at a wavelength of 0.872 Å at 100 K. For selenomethionine-substituted crystals, the ID29 beamline (ESRF Synchrotron, Grenoble)^44^ was used for anomalous diffraction data collection, done at 100 K. The structure was solved by single-wavelength anomalous dispersion (SAD) from a single selenomethionine derivative crystal measured at 0.976 Å, which diffracted up to 2.7 Å. The Auto-Rickshaw program^45^ was then used to phase the protein and build an initial model, which was further manually build and refined using COOT^46^ and phenix.refine from the Phenix package^47^. This model was then used to phase the native data set at a resolution of 2.2 Å. After molecular replacement, automatic model building was performed with the program ARP/wARP^48^, followed by manual iterative cycles of model refinement using the program phenix.refine^47^. Manual adjustments between the refinement cycles were done with the program Coot^46^ and Ramachandran validation was done using MolProbity^49^. Almost all residues (96.3%) were in the preferred regions of the Ramachandran plot, and the remaining 3.7% were in the additionally allowed regions. The data collection and refinement statistics are listed in Table 1. The images of the models were prepared using MacPyMOL^50^.

### IC_50_ determination of nisin and its variants

Cells from the different expressing strains were grown overnight in GM17 media supplemented with 5 μgml^−1^ erythromycin in the presence of 1 ngml^−1^ nisin. The diluted cells (final OD_600_ of 0.1) were incubated with a serial dilution of nisin or its variants in a 96-well plate. The total volume in each well was 200 μl, consisting of 50 μl nisin or its variants and 150 μl GM17 containing the corresponding *L. lactis* strain. The plate was then incubated at 30 °C and after 5 hours, the optical density was measured at 600 nm *via* 96-well plate reader BMG. The IC_50_ value was determined as previously described^51^.

### Molecular dynamics simulations

In order to investigate nisin recognition by *Sa*NSR we performed molecular dynamics (MD) simulations of an unbound *Sa*NSR monomer (NSR_Apo_), a *Sa*NSR monomer bound to the N-terminal part of *Sa*NSR (residues 31–36; in the following named “Tail”) from an adjacent subunit (NSR_Tail_) in the crystal structure (see Fig. 1), and a *Sa*NSR monomer bound to the C-terminal part (residues 22–34; Supplementary Fig. 2c) of nisin (NSR_Nisin_). Initial coordinates for NSR_Apo_ and NSR_Tail_ were taken from the crystal structure described here. Since no structural information is available for nisin bound to *Sa*NSR, we generated models as starting structures for MD simulations by structurally aligning the nisin part to the Tail using the program Moloc. The nisin cleavage site between ring E and Ser_29_ was oriented towards the catalytically active Ser_236_ in *Sa*NSR^22^. Rings D and E were manually placed in three orientations within the binding site such that they showed good complementarity with the *Sa*NSR surface, resulting in three different models of *Sa*NSR/nisin complexes (NSR_Nisin,1_, NSR_Nisin,2_, and NSR_Nisin,3_, Supplementary Fig. 4a).

For the MD simulations, structures of NSR_Apo_, NSR_Tail_, and NSR_Nisin,1-3_ were prepared, relaxed, and thermalized as described in detail in the Supporting Information. Three independent production runs of MD simulations of 500 ns length in the canonical (NVT) ensemble at 300 K were then conducted for each of the five systems, leading to a total simulation time of 5 × 3 × 500 ns = 7.5 μs; see Supporting Information for details.

The trajectories were analyzed with respect to distances, root mean square fluctuations (RMSF) and deviations (RMSD) as a measure for mobility and structural similarity, respectively, and hydrogen bonds defined by a distance between the two donor and acceptor atoms <3.2 Å and an angle (donor atom, H, acceptor atom) between 120° and 180° using *cpptraj*^52^. Salt-bridge interactions are defined by a distance <4.0 Å between the center of mass of both charged groups. The set of structural models binding to *Sa*NSR (see section “Structural model of nisin binding to *Sa*NSR) was generated by structurally aligning *Sa*NSR and subsequent RMSD calculations for the nisin peptide.

### Calculation of the effective binding energy

In order to identify amino acids in *Sa*NSR that contribute most to nisin binding, we computed the residue-wise contribution to binding effective energies by the “single trajectory” molecular mechanics Poisson-Boltzman area (MM-PBSA) approach^53^,^54^,^55^. To determine the per-residue contribution, the decomposition scheme^56^ as implemented in the mm_pbsa.pl script in Amber 14^37^ was applied. The calculations were performed with the ff99SB force field^57^,^58^. The polar part of the solvation free energy was determined by applying the PBSA solver using a dielectric constant of 1 (solute) and 80 (solvent) together with Parse radii^59^. The conformational ensemble consists of 10,000 snapshots and was extracted from the 1–200 ns interval of each of the NSR_Nisin,1-2_ trajectories. Prior to the MM-PBSA computations, counter ions and water molecules were stripped from the snapshots. For the computations, we considered the *Sa*NSR protein the receptor, whereas the nisin C-terminus was considered the ligand. All residues in *Sa*NSR and nisin were considered for per-residue decomposition. Rings D and E in nisin were treated as one residue.

## PDB Deposition

The final model has been deposited in the PDB database under the accession code: 4Y68.

## Additional Information

**How to cite this article**: Khosa, S. *et al.* Structural basis of lantibiotic recognition by the nisin resistance protein from *Streptococcus agalactiae*. *Sci. Rep.* **6**, 18679; doi: 10.1038/srep18679 (2016).

## Supplementary Material

Supplementary Information

## Figures and Tables

**Table 1:** Data collection, phasing and refinement statistics for *Sa*NSR.

	Native *Sa*NSR	SeMet *Sa*NSR
**Data collection**		
Space group	*P*2_1_2_1_2_1_	*P*4_3_2
Cell dimensions		
a, b, c (Å)	58.8, 137.2, 164.0	186.1, 186.1, 186.1
α, β, γ (°)	90, 90, 90	90, 90, 90
Wavelength	0.87260	0.97625
Resolution (Å)	100.0–2.21 (2.29–2.21)	100.0–2.80 (2.9-2.8)
R_merge_	11.5 (63.8)	29.3 (110.5)
<I / σ (I)>	8.67 (1.79)	20.33 (1.72)
Completeness (%)	99.6 (99.3)	99.8 (98.7)
Redundancy	4.5 (4.2)	75.6 (70.5)
**Refinement**		
Resolution (Å)	55.36-2.21 (2.28-2.21)	
No. reflections	303208 (27954)	
R_work_/R_free_	0.19 (0.27)/ 0.24 (0.31)	
*No. of atoms*	9588	
Protein	9017	
Ligand/ion	48	
Water	523	
*B*-factors (Å^3^)	40.5	
Protein	40.3	
Ligand/ion	68.1	
Water	41.2	
*R.m.s deviations*		
Bond lengths (Å)	0.008	
Bond angles (°)	1.09	

^*^Values in parentheses are for highest-resolution shell.
