# Supplementary material for: Structural basis of lantibiotic recognition by the nisin resistance protein from Streptococcus agalactiae
Source: Sci Rep. 2016 Jan 4;6:18679. doi: 10.1038/srep18679 (PMC4698656; doi:10.1038/srep18679)
Supplement: Supplementary Information [file srep18679-s1.pdf]

Supplementary information

Structural basis of lantibiotic recognition by the nisin resistance protein from *Streptococcus agalactiae*

Sakshi Khosa[a#], Benedikt Frieg[b#], Daniel Mulnaes[b] Diana Kleinschrodt[c], Astrid Hoeppner[d], Holger Gohlke[b] and Sander H.J. Smits[a]*

[a]Institute of Biochemistry, Heinrich Heine University, Universitätsstr. 1, 40225 Düsseldorf, Germany
[b]Institute of Pharmaceutical and Medicinal Chemistry, Heinrich Heine University, Universitätsstr. 1, 40225 Düsseldorf, Germany
[c]Protein Production Facility, Heinrich Heine University, Universitätsstr. 1, 40225 Düsseldorf, Germany
[d]Crystal and X-ray Facility, Heinrich Heine University, Universitätsstr. 1, 40225 Düsseldorf, Germany

[#]Both authors contributed equally.
*Corresponding author: Phone: +49-211-81-12647; Fax: +49-211-81-15310; E-Mail: sander.smits@hhu.de

Material and Methods

Cloning, expression and purification of *Sa*NSR

The primers were designed in such a way that the first 30 amino acids encoding for the transmembrane helix were not present in the construct. This allowed soluble expression and included an 8xhis-tag at the N-terminus for purification purposes. *Sa*NSR was expressed and purified via two-step purification protocol. A single transformed colony was inoculated into 20 ml LB media containing 30 μg ml^{-1} kanamycin. The culture was grown for 14 h at 310 K with shaking at 200 rev min^{-1}. 2 L LB media with 30 μg ml^{-1} kanamycin was inoculated with the overnight culture at an OD600 of 0.05 and grown at 310 K with shaking at 170 rev min^{-1} till OD_{600} of 0.3 was reached. The temperature was lowered to 291 K and the cells were further grown till OD_{600} of 0.8 before induction with 1 mM IPTG. The cells were further grown for 15 h.

The cells were harvested by centrifugation at 8000 rev min^{-1} for 20 min at 277 K. The harvested cell pellet was stored at 253 K till further use. The stored cell pellet was thawed and resuspended in 10 ml of buffer A (50 mM Tris pH 8.0, 50 mM NaCl and 10% glycerol) and 10 mg of DNase (Deoxyribonuclease I from bovine pancreas, Sigma Aldrich) was added. The cells were lysed five times using a cell disruptor (Constant Cell Disruption Systems, United Kingdom) at 37709 psi (1kbar = 14.50 psi). The lysate was centrifuged at 42000 rev min^{-1} for 60 min using a Ti60 rotor to remove unlysed cells and debris.

Histidine was added to the cleared lysate at a final concentration of 1 mM. The lysate was then applied to a Ni^{2+} loaded HiTrap HP Chelating column (GE Healthcare) pre-equilibrated with buffer B (20 mM Tris pH 8.0, 250 mM NaCl and 1 mM Histidine) at a flow rate of 1 ml min^{-1}. The column was washed with six column volumes of buffer B. The protein was then eluted with increasing concentrations of Histidine from 1 mM to 120 mM, in form of a linear gradient spanning 60 min with a flow rate of 2 ml min^{-1}. The fractions containing the protein of interest were pooled and concentrated up to 12 mg ml^{-1} in an Amicon centrifugal filter concentrator with a 10 kDa cut-off membrane (Millipore). The concentrated protein was then further purified by size exclusion chromatography using Superose 12 GL 10/300 column (GE Healthcare), equilibrated with buffer C (25 mM MES pH 6.0, 150 mM NaCl). The protein eluted as a single homogeneous peak and the concerned fractions were pooled and concentrated to 8.6 mg ml^{-1} as mentioned before. The purity of the protein was analyzed with SDS-PAGE and colloidal coomassie stain.

To determine the oligomeric state of *Sa*NSR protein in solution, we used conventional size-exclusion chromatography (SEC) and high performance liquid chromatography coupled to multi angle light scattering detection (HPLC-MALS). SEC was performed as described previously[1] and the size-exclusion column was standardized with a gel filtration markers kit (Sigma).

Crystallization

Crystallization screening was performed at 285 K using NT8 robot (Formulatrix) and sitting-drop vapour diffusion method in Corning 3553 sitting drop plates. For initial screening different commercial crystallization screens were used {Nextal JCSG Core Suites I, Classics Suite, PEGs Suite, MPD Suite (Qiagen, Germany) and MIDAS (Molecular Dimensions, England)}. Nanodrops consisting of 0.1 µl each of protein and reservoir solution were mixed and equilibrated over 50 µl reservoir solution. The screening yielded some initial rod shaped crystals after three days in the condition 0.5 M lithium sulfate and 15% (w/v) PEG 8000 (Classic I suite, condition F5). The initial crystals were optimized by varying the concentration of PEG (5, 10, 15, 20, 25 and 30% (w/v)) and salt (0.4, 0.5, 0.6 and 0.7 M), using hanging and sitting-drop vapour diffusion methods at 297 K and 285 K, respectively. Each drop consisted of 1 µl of protein solution (concentration of 9 mg ml^{-1}) mixed with 1 µl of reservoir solution, equilibrated over a reservoir volume of 500 µl. Crystals were obtained after one day and grew to their maximum dimensions within 5 days. For preliminary analysis of the crystals see [1].

Expression, purification and crystallization of selenomethionine-substituted SaNSR

For selenomethionine substitution, *E. coli* BL834 (DE3) cells were grown according to manufacturer's protocol in M9 minimal media (Molecular Dimensions) supplemented with 50 µg ml^{-1} of L-seleno-methionine. Expression and purification were identical to the native SaNSR [1]. Selenomethionine derivatized *Sa*NSR was crystallized in a similar manner as the native protein, using the hanging drop vapor diffusion method with a protein concentration of 10 mg ml^{-1}.

Cloning of pNZ-SV-*Sa*NSR and variants

The plasmid pNZ-SV-*Sa*NSR (N-His) was cloned with the In-Fusion HD PCR Cloning Kit (Clontech) as previously published [2]. Different mutations were introduced into the pNZ-SV-

*Sa*NSR (N-His) using standard site-directed mutagenesis protocol. The used primers are listed in Supplementary Table 1a.

Expression of *Sa*NSR and its variants in *L. lactis* NZ9000

The plasmid encoding pNZ-SV-*Sa*NSR and its variants were transformed into the nisin sensitive *L. lactis* strain NZ9000. As a control the empty vector was also transformed, termed NZ9000Erm. The strains expressing NZ9000SaNSR and its mutations were grown in GM17 media supplemented with 5 µg ml^{-1} erythromycin to an OD_{600} of 0.8. The expression was induced by the addition of nisin (at a final concentration of 1 $ngml^{-1}$) and the cultures were further grown overnight. The cells were then diluted to an OD_{600} of 0.1 in fresh GM17 media supplemented with 5µg ml^{-1} erythromycin. These cells were then used for the assays described below.

Cloning, Overexpression and purification of nisin and its variants

Nisin was purified from commercially available powder as described [3]. The cloning, overexpression and purification of precursor nisin variants were performed as described previously [3,4], excepting that the elution buffer of the cationic exchange chromatography of the various precursor nisin variants were changed to 50 mM HEPES-NaOH, pH 7.0, 1 M NaCl, and 10% glycerol. The concentrations of nisin and its variants were determined by using RP-HPLC and in order to activate the nisin variants, the leader peptide was cleaved off using the protease NisP as previously described, thereby [5].

Molecular dynamics simulations

Structures of NSR_{Apo}, NSR_{Tail}, and $NSR_{Nisin,1-3}$ were prepared using LEaP[6] of the Amber 14 suite of programs [7]. First, missing hydrogen atoms were added by LEaP [6], and histidine residues were assigned the HIE state. Second, counter ions were added to neutralize each system. Finally, systems were solvated using the TIP3P water model [8]. The obtained systems comprised ~ 60.000 atoms. Atomic partial charges for Dha33 (dehydroalanine) and rings D and E in nisin, which are treated as one "residue" in the Amber scheme, were obtained following the RESP procedure[9] using Gaussian09 [10]. For the non-standard amino acid Dha33, force field parameters were adapted from ref. [11]. All other parameters were taken from the Amber ff99SB force field [12,13]. Structural relaxation, thermalization, and

production runs of MD simulations were conducted with pmemd.cuda [14] of Amber 14 [7]. Two steps of energy minimization were performed to relax the systems. First, harmonic restraints with a force constant of 25 kcalmol^{-1}Å^{-2} were applied to all protein atoms while all other atoms were free to move during 50 cycles of steepest descent (SD) and 200 cycles of conjugate gradient (CG) minimization. Second, the force constant of the harmonic restraints was reduced to 5 kcalmol^{-1}Å^{-2}, and 50 cycles of SD and 200 cycles of CG minimization were performed. Subsequently, the systems were heated from 100 K to 299.9 K, 300 K, or 300.1 K during canonical (NVT) MD simulations of 50 ps length to setup three independent MD production simulations for NSR_{Apo}, NSR_{Tail}, and $NSR_{Nisin,1-3}$, respectively. Afterwards, the density was adjusted to 1 g·cm-3 during 30 ps of isobaric-isothermal (NPT) MD simulations. During heating and density adaptation, positional restraints of 5 kcal·mol^{-1}·Å^{-2} were applied to all protein atoms. Finally, these positional restraints were removed by gradually decreasing the force constant from 5 to 0 kcal·mol^{-1}·Å^{-2} in six NVT-MD runs of 10 ps length each. For MD simulations, the particle mesh Ewald (PME) method [15-17] was employed to treat long-range electrostatic interactions. For short-range non-bonded interactions, we set a distance cutoff of 8 Å. The SHAKE algorithm [18] was applied to all bonds involving hydrogens, allowing a 2 fs time step for integrating Newton's equations of motion. Production MD simulations were performed in the NVT ensemble at 300 K for 500 ns. Coordinates were saved every 20 ps and used for analyses. This led to a total simulation time of 5 x 3 x 500 ns = 7.5 µs.

Supplementary Table 1

(a) IC_{50} values of nisin and its variants against the NZ9000Erm and NZ9000-*Sa*NSR strains as well as the calculated "Fold of resistance".

	**NZ9000Erm (nM)**	**NZ9000-*Sa*NSR (nM)**	***Fold of resistance***
**Nisin**	3.3±0.1	66.4±2.1	20.1
**CCCCA**	42.2±0.7	57.8±3.3	1.7
**CCCAA**	184.9±9.7	259.5±16.6	1.4
**$Nisin_{1-22}$**	294.7±9.7	121.1±6.1	0.4
**$Nisin_{1-28}$**	277.1±14.0	103.5±5.5	0.4

The values reported are the average over minimum triplicates ± SEM.

(b) IC_{50} values of nisin against the NZ9000Erm, NZ9000-*Sa*NSR, and NZ9000-*Sa*NSR variant strains.

	IC_{50} value (nM)	*Activity (%)*
NZ9000Erm	3.3±0.1	0
NZ9000-*Sa*NSR (wildtype)	66.4±2.1	100
NZ9000-*Sa*NSR-His_{98}Ala	12.3±1.5	14.2
NZ9000-*Sa*NSR-Ser_{236}Ala	12.6±0.7	14.6
NZ9000-*Sa*NSR-Ser_{237}Ala	50.2±2.3	74.3
NZ9000-*Sa*NSR-Glu_{239}Ala	17.1±0.7	21.7
NZ9000-*Sa*NSR-Asn_{172}Ala	33.5±2.9	47.7
NZ9000-*Sa*NSR-Met_{173}Ala	30.3±1.4	42.7
NZ9000-*Sa*NSR-Ile_{174}Ala	24.1±2.2	32.9
NZ9000-*Sa*NSR-Thr_{263}Ala	16.0±0.3	20.1
NZ9000-*Sa*NSR-Asn_{265}Ala	22.1±1.1	29.7
NZ9000-*Sa*NSR-Thr_{267}Ala	48.5±0.6	71.6
NZ9000-*Sa*NSR-Asp_{110}Ala	32.8±2.1	46.7
NZ9000-*Sa*NSR-Arg_{275}Ala	33.6±2.3	47.9

The values reported are the average over minimum triplicates ± SEM.

Supplementary Table 2: Primers used in this study.

Shown below are the primers used to create the point mutations within the expression plasmid pNZ-SV-*Sa*NSR-NHis [1] .

Primer Name	***Sequence (5'-3')***
His_{98}Ala-for	CGGTATGGAGGAGGTAAAGCAAGTCAAATATTATCC
His_{98}Ala-rev	GGATAATATTTGACTTGCTTTACCTCCTCCATACCG
Ser_{236}Ala-for	CTAATCATAAAACTGCTGCGTCGGCAGAAATGAC
Ser_{236}Ala-rev	GTCATTTCTGCCGACGCAGCAGTTTTATGATTAG
Ser_{237}Ala-for	CTAATCATAAAACTGCTAGTGCAGCAGAAATGACTTTTTATC
Ser_{237}Ala-rev	GATAAAAAAGTCATTTCTGCTGCACTAGCAGTTTTATGATTAG
Glu_{239}Ala-for	CTGCTAGTTCGGCAGCAATGACTTTTTTATC
Glu_{239}Ala-rev	GATAAAAAAGTCATTGCTGCCGAACTAGCAG
Asn_{172}Ala-for	CGAATAATACTGGCGGCGCAATGATCCCTATGATTG
Asn_{172}Ala-rev	CAATCATAGGGATCATTGCGCCGCCAGTATTATTCG
Met_{173}Ala-for	GAATAATACTGGCGGCAATGCAATCCCTATGATTGGG
Met_{173}Ala-rev	CCCAATCATAGGGATTGCATTGCCGCCAGTATTATTC
Ile_{174}Ala-for	CTGGCGGCAATATGGCACCTATGATTGGGGG
Ile_{174}Ala-rev	CCCCCAATCATAGGTGCCATATTGCCGCCAG
Thr_{263}Ala-for	CAGCAGGATATACGGCAGTTAATGAAACTTTC
Thr_{263}Ala-rev	GAAAGTTTCATTAACTGCCGTATATCCTGCTG
Asn_{265}Ala-for	GCAGGATATACGACTGTTGCAGAAACTTTCATGCTTTAC
Asn_{265}Ala-rev	GTAAAGCATGAAAGTTTCTGCAACAGTCGTATATCCTGC
Thr_{267}Ala-for	GATATACGACTGTTAATGAAGCATTCATGCTTTACGACG
Thr_{267}Ala-rev	CGTCGTAAAGCATGAATGCTTCATTAACAGTCGTATATC
Asp_{110}Ala-for	GAGACTGTACGAAGAGCAACCCTAGATAGTCG
Asp_{110}Ala-rev	CGACTATCTAGGGTTGCTCTTCGTACAGTCTC
Arg_{275}Ala-for	GCTTTACGACGGTGCTGCATTAGCTTTAACTACAG
Arg_{275}Ala-rev	CTGTAGTTAAAGCTAATGCAGCACCGTCGTAAAGC

Supplementary Figure 1: Schematic representation of the structure of nisin and the different variants used in this study.

Shown are structural representations of wildtype nisin and the variants CCCCA, CCCAA, nisin$_{1-22}$ and nisin$_{1-28}$. Highlighted in yellow are the dehydrated amino acids while the amino acids which are dehydrated as well as cyclized are shown in orange. The lanthionine rings are also shown in orange and are numbered A-E.

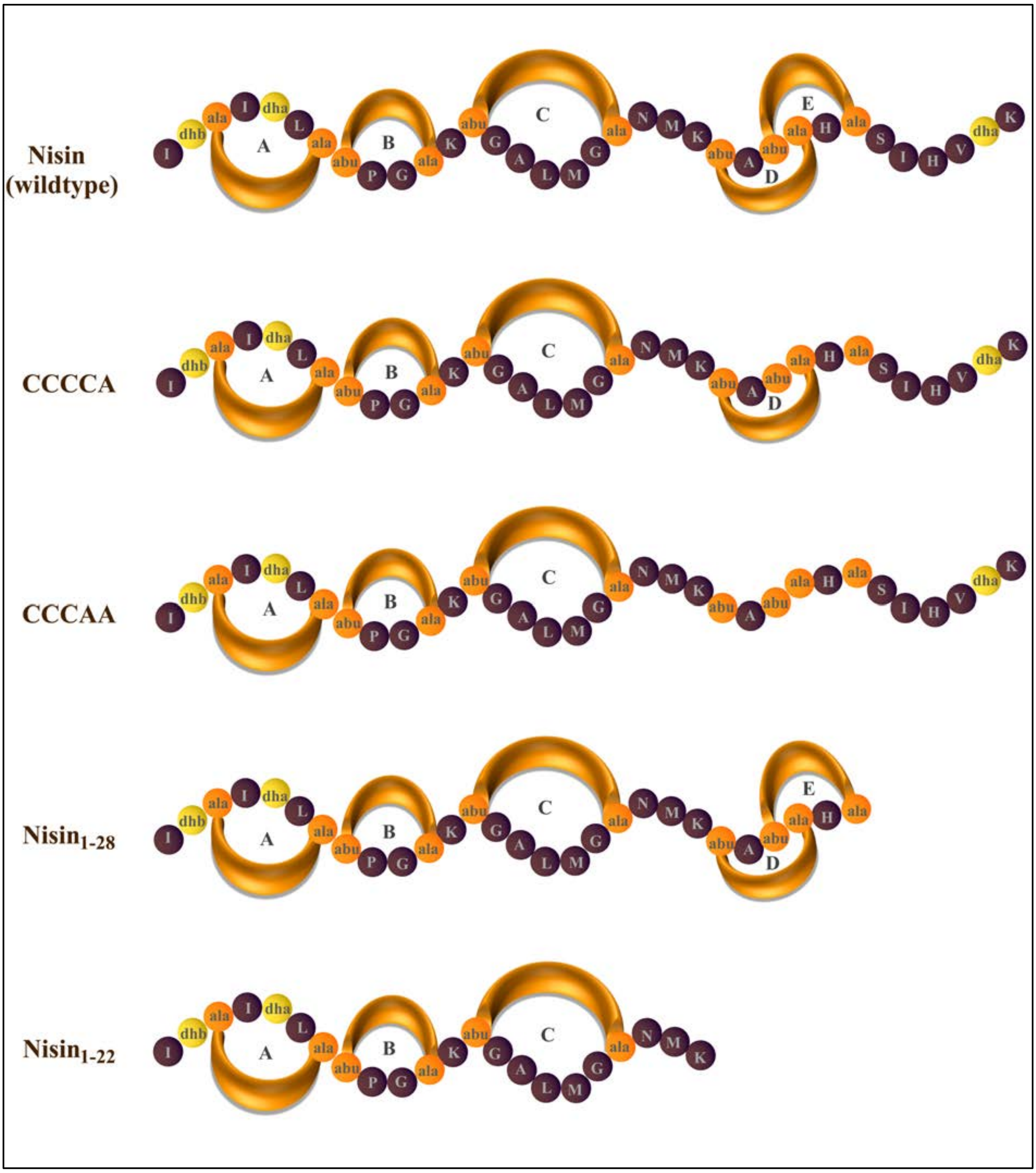

Supplementary Figure 2: Purification, oligomeric state and structure of *Sa*NSR.

(a) 15% SDS gel showing purified *Sa*NSR after the two-step purification involving IMAC and SEC. Lane M represents PageRuler Unstained Protein Ladder; the remaining lanes are the purified *Sa*NSR fractions at 35 kDa. The lower 30 kDa band also arises from *Sa*NSR, as verified by mass spectrometry, and could likely be a degradation product. (b) Determination of the oligomeric state of the purified *Sa*NSR protein using HPLC-MALS. The x-axis represents the time in minutes; the left and right y-axes depict the relative intensity and molecular mass, respectively. The blue line is the differential refractive index signal; the blue dotted line indicates the calculated molar mass. (c) The structure of *Sa*NSR in the asymmetric unit. The four copies of the monomer are colored in green, cyan, pink and yellow for chains A, B, C and D, respectively.

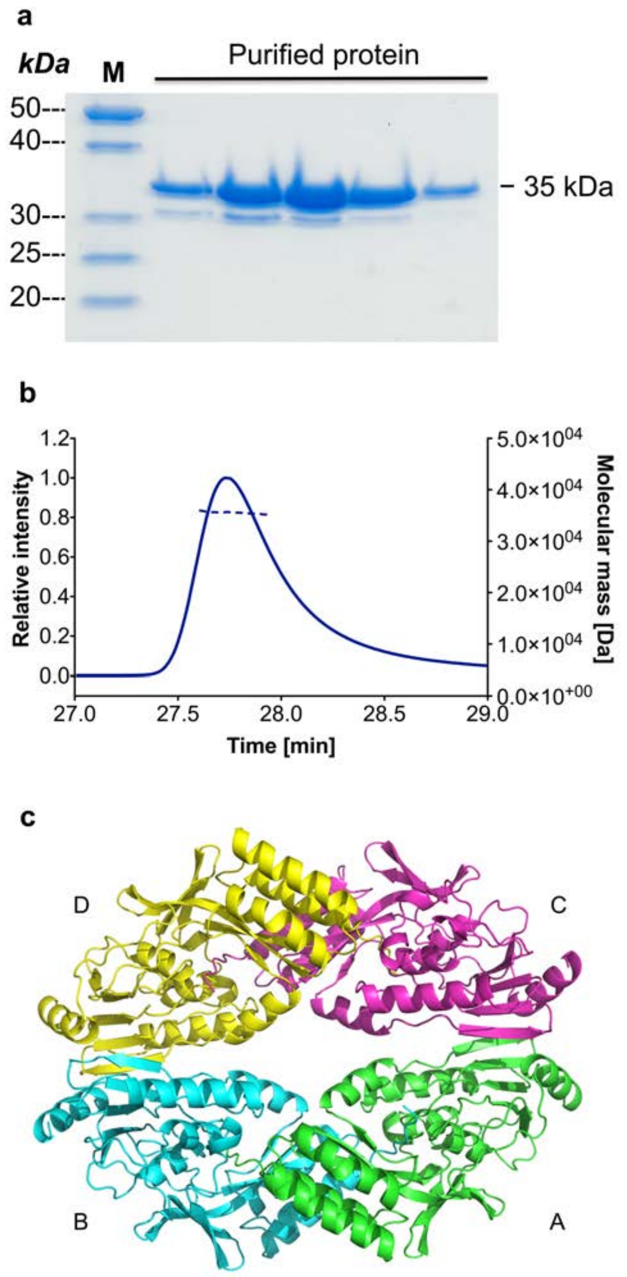

Supplementary Figure 3: Alignment of the nisin resistance proteins.

Homolog sequences of nisin resistance protein were aligned using ClustalW2 [2]. Visualization of the sequence alignment was performed by ESPript [3]. Secondary structure elements were calculated based on the *Sa*NSR structure. The TASSAEM motif is marked by a dotted blue line.

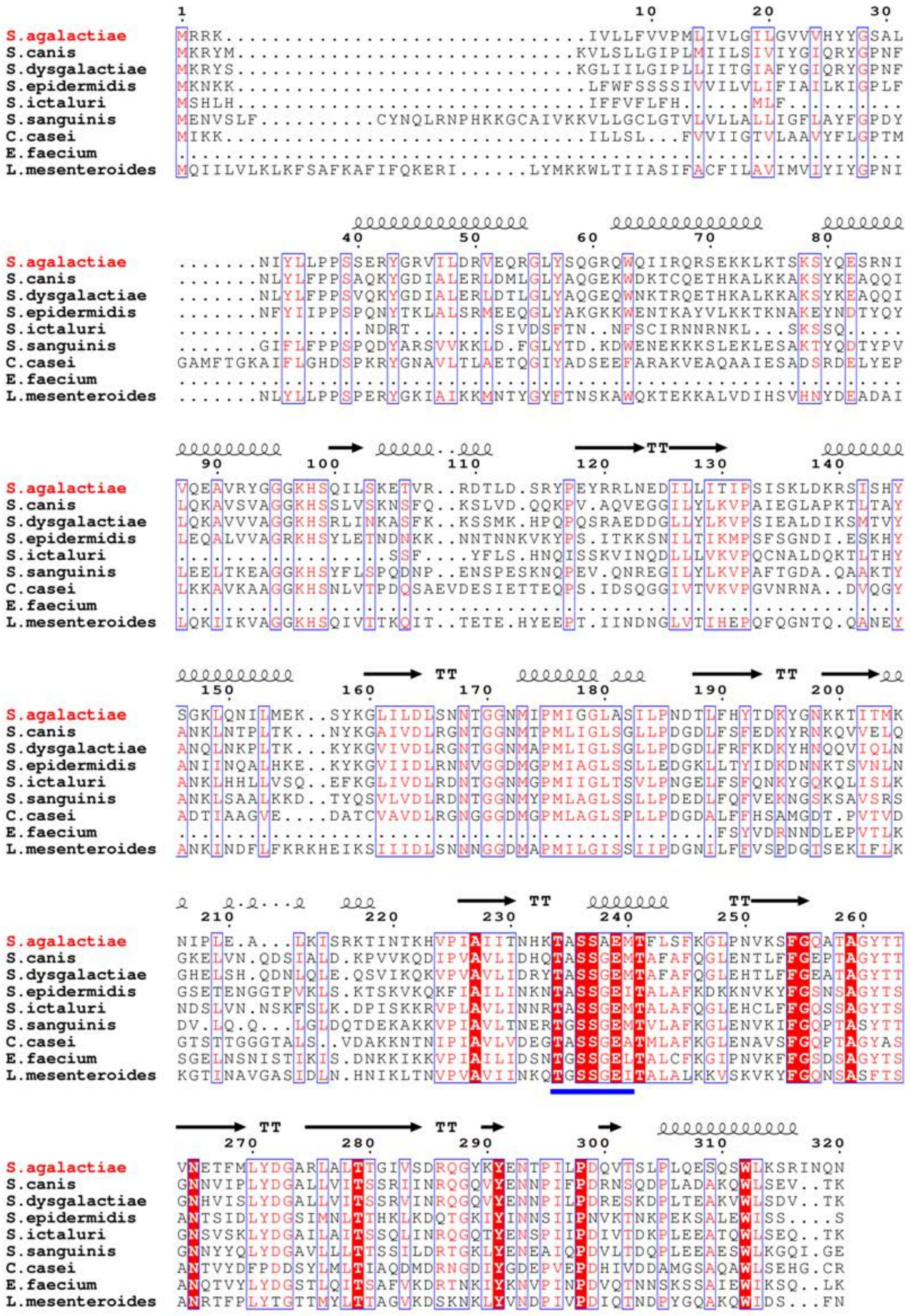

Supplementary Figure 4: Mobility and structural deviations in MD simulations of NSR_{Apo} and NSR_{Tail}.

(a) Mean backbone RMSF of residues of the N-terminal helical bundle in NSR_{Apo} models after superimposition of the cap domain and the protease core for three trajectories (colored differently) of 500 ns length each. (b) Backbone RMSD for N-pep in the NSR_{Tail} model for three independent MD simulations. Mean values are shown in the legend. Mean standard error < 0.1 Å and not shown.

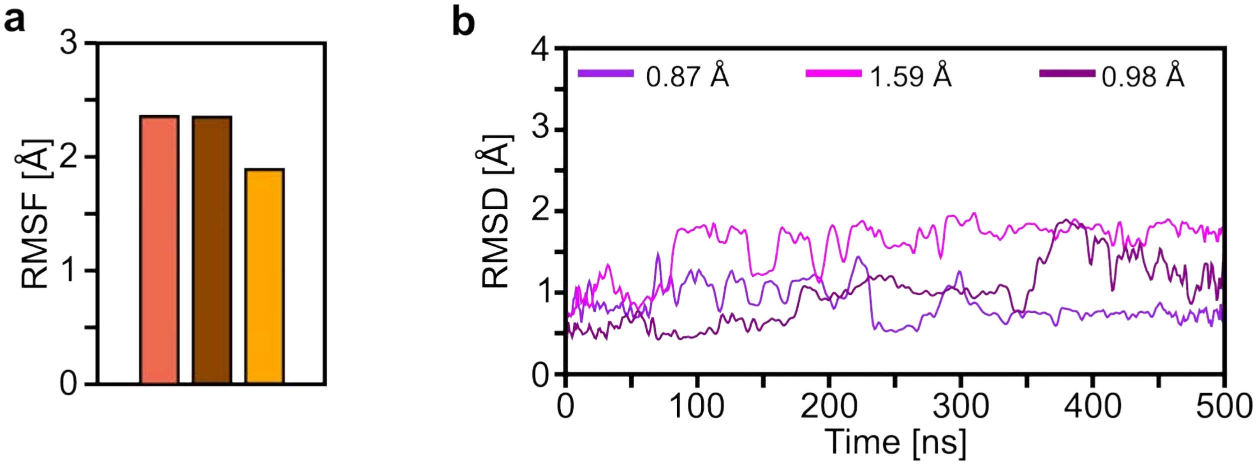

Supplementary Figure 5: Models of *Sa*NSR/nisin complexes used as starting structures for and RMSD analysis of MD simulations.

(a) View into the binding site of *Sa*NSR with Asn_{172}, Met_{173}, and Ile_{174} colored in magenta, Ser_{236} in blue, and Tyr_{261} in orange; for clarity, the N-terminal bundle and protease core domains of *Sa*NSR were omitted. The C-terminus of nisin (residues 22 - 34) is shown in ball and stick representation. The right panels show a close-up view of all initial NSR/nisin complexes used as starting structures for the MD simulations (red: $NSR_{Nisin,1}$; green: $NSR_{Nisin,2}$; blue: $NSR_{Nisin,3}$).

(b) All atom RMSD values, relative to the respective average structures, over 500 ns of MD simulations for the C-terminus of nisin and for the $Nisin_{core}$ (composed of rings D and E, Ser_{29}, and Ile_{30}; see Figure 4b-d). RMSD values are shown for each of the three independent MD trajectories.

a

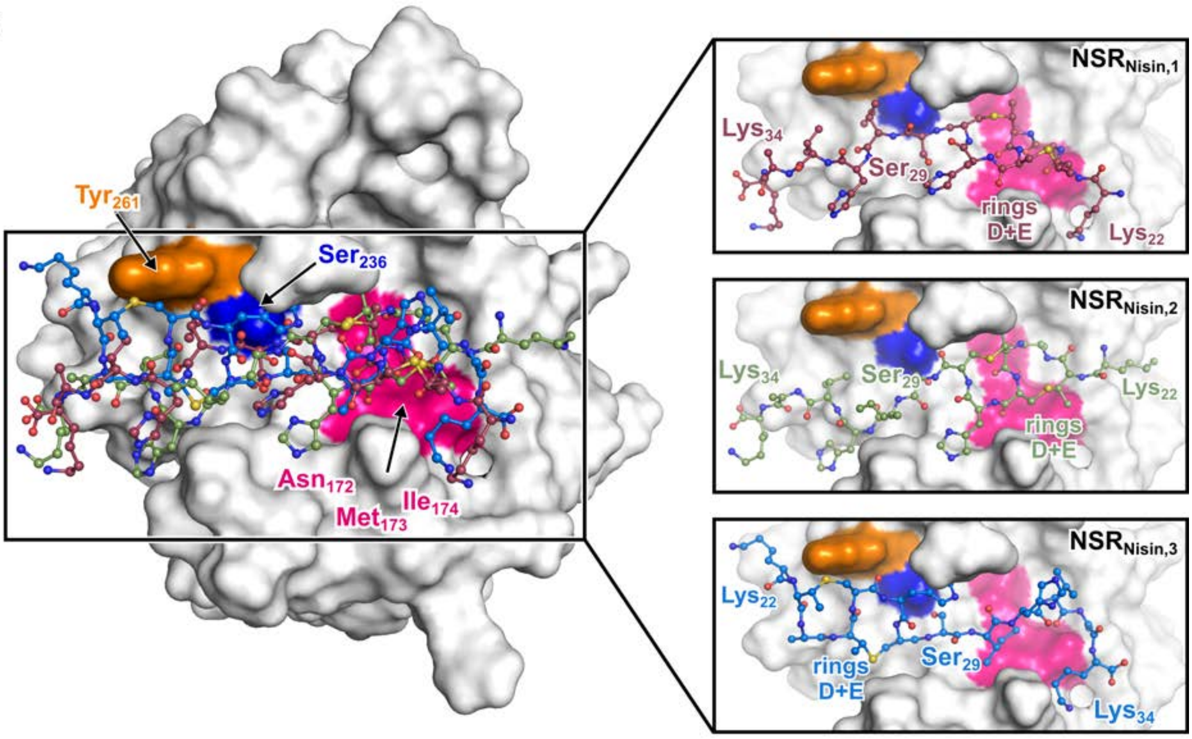

b

Model \ RMSD [Å][a]	mean $Nisin_{C\text{-}terminus}$[b]	mean $Nisin_{core}$[b]
$NSR_{Nisin,1}$ (1)	2.75	1.38
$NSR_{Nisin,1}$ (2)	2.60	1.34
$NSR_{Nisin,1}$ (3)	3.19	2.27
$NSR_{Nisin,2}$ (1)	1.44	0.80
$NSR_{Nisin,2}$ (2)	1.80	1.34
$NSR_{Nisin,2}$ (3)	2.16	1.28

[a] relative to average structure
[b] MSE < 0.01 Å

Supplementary Figure 6: Structural analyses of the TASSAEM region over the MD trajectories.

(a) Close-up view of the catalytic site residues in blue and Gly_{171} from the protease core in magenta ball-and-stick representation. Identified hydrogen bonds (1 – 7) are depicted as orange dotted lines. The table shows minimum (min.) and maximum (max.) occupancies of hydrogen bonds 1 to 7 across the six MD trajectories. Distance between the side chain oxygen in Ser_{237} and the carbonyl carbon of ring E from the nisin cleavage site (b), and the side chain oxygen in Ser_{237} and the δ-nitrogen in His_{98} (c) for both $Nsr_{Nisin,1}$ (top panel) and $Nsr_{Nisin,2}$ (bottom panel) models during the three independent MD simulations. Mean values over each trajectory are shown in the legend.

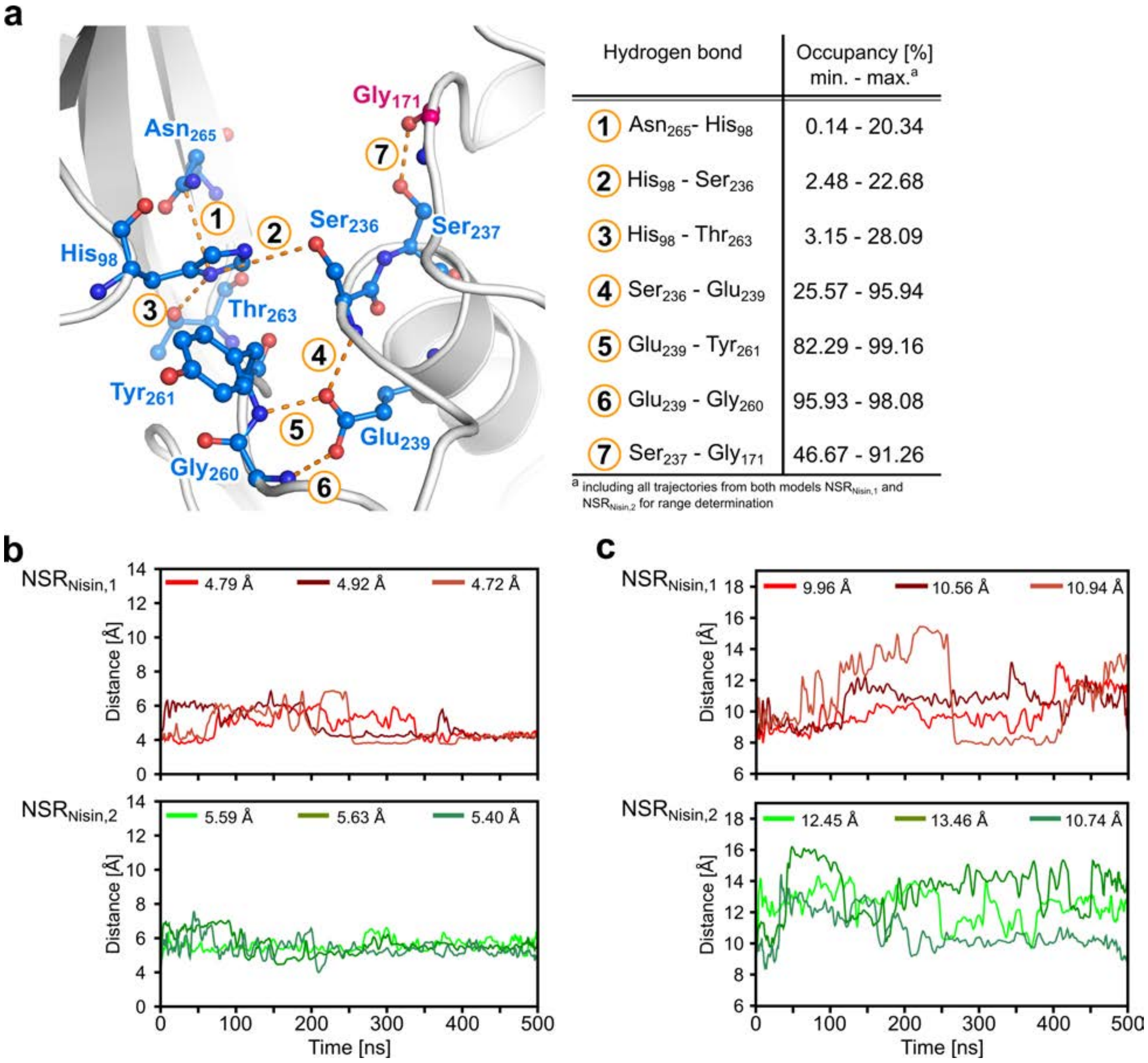

Hydrogen bond	Occupancy [%] min. - max.[a]
1 Asn_{265} - His_{98}	0.14 - 20.34
2 His_{98} - Ser_{236}	2.48 - 22.68
3 His_{98} - Thr_{263}	3.15 - 28.09
4 Ser_{236} - Glu_{239}	25.57 - 95.94
5 Glu_{239} - Tyr_{261}	82.29 - 99.16
6 Glu_{239} - Gly_{260}	95.93 - 98.08
7 Ser_{237} - Gly_{171}	46.67 - 91.26

[a] including all trajectories from both models $NSR_{Nisin,1}$ and $NSR_{Nisin,2}$ for range determination

Supplementary Figure 7: Energetic and structural analyses of nisin binding over the MD trajectories.

(a) Per-residue effective binding energies computed by the MM-PBSA approach for residues within 5 Å of the C-terminus of nisin occupying the catalytic site of *Sa*NSR for model $NSR_{Nisin,1}$ (red) and $NSR_{Nisin,2}$ (green). The gray lines indicate a threshold of ± 0.8 kcal mol^{-1}. (b) Close-up view of the binding site residues (magenta) in the crystal structure bound to nisin (orange). Identified hydrogen bonds (1 – 3) are depicted as orange dotted lines. (c) Distance between the side chain carboxylate of residue Glu_{266} and the guanidino group of Arg_{54} from the N-terminal helical bundle for both $NSR_{Nisin,1}$ (top panel) and $NSR_{Nisin,2}$ (bottom panel) over the course of 500 ns MD simulations. Mean values for each MD trajectory are shown in the legend. (d) Close-up view of the binding site residues (magenta) and nisin (orange). Water-mediated interactions (1 – 2) are indicated by dark blue dotted lines. In (b) and (d), the tables show minimum and maximum occupancies of hydrogen bonds or water-mediated interactions. Hydrogen bonds and water-mediated interactions were determined for each of the $NSR_{Nisin,1}$ and $NSR_{Nisin,2}$ trajectories.

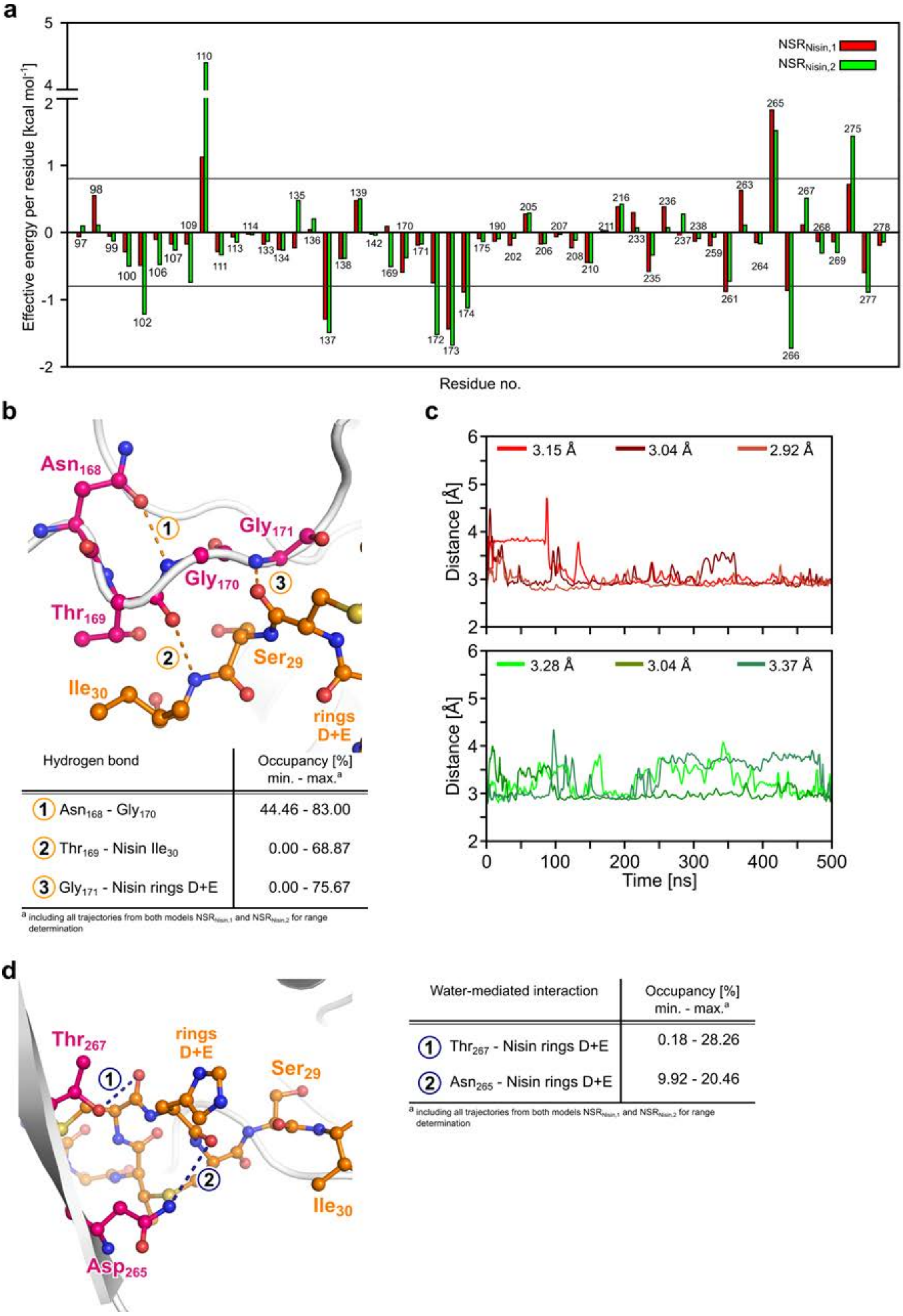

Hydrogen bond	Occupancy [%] min. - max.[a]
(1) Asn_{168} - Gly_{170}	44.46 - 83.00
(2) Thr_{169} - Nisin Ile_{30}	0.00 - 68.87
(3) Gly_{171} - Nisin rings D+E	0.00 - 75.67

[a] including all trajectories from both models $NSR_{Nisin,1}$ and $NSR_{Nisin,2}$ for range determination

Water-mediated interaction	Occupancy [%] min. - max.[a]
(1) Thr_{267} - Nisin rings D+E	0.18 - 28.26
(2) Asn_{265} - Nisin rings D+E	9.92 - 20.46

[a] including all trajectories from both models $NSR_{Nisin,1}$ and $NSR_{Nisin,2}$ for range determination

Supplementary Figure 8: Structural analysis of helix α4 over the MD trajectories.

(a) For models $NSR_{Nisin,1}$, and $NSR_{Nisin,2}$ distances were measured between the side chain carboxylate of Asp_{110} and the guanidino group of Arg_{275}. (b) Residue-wise α-helix probability for residues that compose helix α4. Mean distances and mean α-helix probabilities over the independent MD trajectories are shown in the legend.

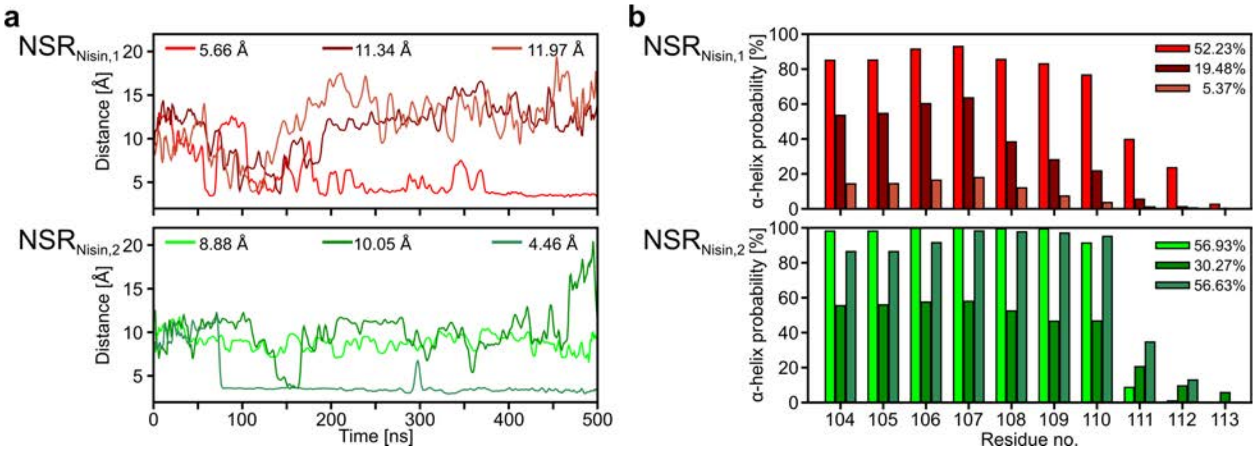

Supplementary References

1. Khosa, S., Hoeppner, A., Kleinschrodt, D. & Smits, S. Overexpression, purification, crystallization and preliminary X-ray diffraction of the nisin resistance protein from *Streptococcus agalactiae*. *Acta Crystallogr. F Biol. Crystallogr.* **71**, 671-675 (2015).

2. Khosa, S., Alkhatib, Z. & Smits, S.H. NSR from *Streptococcus agalactiae* confers resistance against nisin and is encoded by a conserved nsr operon. *Biol. Chem.* **394**, 1543-1549 (2013).

3. Abts, A. et al. Easy and Rapid Purification of Highly Active Nisin. *International Journal of Peptides* **2011**, 9 (2011).

4. Mavaro, A. et al. Substrate recognition and specificity of the NisB protein, the lantibiotic dehydratase involved in nisin biosynthesis. *J. Biol. Chem.* **286**, 30552-30560 (2011).

5. Abts, A., Montalban-Lopez, M., Kuipers, O.P., Smits, S.H. & Schmitt, L. NisC binds the FxLx motif of the nisin leader peptide. *Biochemistry* **52**, 5387-5395 (2013).

6. Zhang, W., Hou, T., Schafmeister, C., Ross, W.S. & Case, D.A. LEaP. *University of California, San Francisco* (1995).

7. Case, D.A. et al. AMBER 14. *University of California, San Francisco.* (2014).

8. Jorgensen, W.L., Chandrasekhar, J., Madura, J.D., Impey, R.W. & Klein, M.L. Comparison of simple potential functions for simulating liquid water. *J. Chem. Phys.* **79**, 926-935 (1983).

9. Bayly, C.I., Cieplak, P., Cornell, W.D. & Kollman, P.A. A well-behaved electrostatic potential based method using charge restraints for deriving atomic charges - the RESP Model. *J. Phys. Chem.* **97**, 10269-10280 (1993).

10. Frisch, M.J. et al. Gaussian 09. (Gaussian, Inc., Wallingford, CT, USA, 2009).

11. Alagona, G. & Ghio, C. Force field parameters for molecular mechanical simulation of dehydroamino acid residues. *J. Comput. Chem.* **12**, 934-942 (1991).

12. Cornell, W.D. et al. A second generation force field for the simulation of proteins, nucleic acids, and organic molecules. *J. Am. Chem. Soc.* **118**, 2309-2309 (1996).

13. Hornak, V. et al. Comparison of multiple Amber force fields and development of improved protein backbone parameters. *Proteins: Struct., Funct., Bioinf.* **65**, 712-725 (2006).

14. Salomon-Ferrer, R., Gotz, A.W., Poole, D., Le Grand, S. & Walker, R.C. Routine microsecond molecular dynamics simulations with Amber on GPUs. 2. Explicit solvent particle mesh ewald. *J. Chem. Theory Comput.* **9**, 3878-3888 (2013).

15. York, D.M., Darden, T.A. & Pedersen, L.G. The effect of long-range electrostatic interactions in simulations of macromolecular crystals - a comparison of the ewald and truncated list methods. *J. Chem. Phys.* **99**, 8345-8348 (1993).

16. Darden, T., York, D. & Pedersen, L. Particle mesh Ewald: An N· log (N) method for Ewald sums in large systems. *J. Chem. Phys.* **98**, 10089-10092 (1993).

17. Srinivasan, J., Cheatham, I., T. E., Cieplak, P., Kollman, P.A. & Case, D.A. Continuum solvent studies of the stability of DNA, RNA, and phosphoramidate - DNA helices. *J. Am. Chem. Soc.* **120**, 9401-9409 (1998).

18. Ryckaert, J.P., Ciccotti, G. & Berendsen, H.J.C. Numerical integration of cartesian equations of motion of a system with constraints molecular dynamics of n-alkanes. *J. Comput. Phys.* **23**, 327-341 (1977).

19. Larkin, M.A. et al. Clustal W and Clustal X version 2.0. *Bioinformatics* **23**, 2947-2948 (2007).

20. Robert, X. & Gouet, P. Deciphering key features in protein structures with the new ENDscript server. *Nucleic Acids Res.* **42**, W320-W324 (2014).
